# NheABC is a pH-dependent cytotoxin that targets mitochondria and contributes to the virulence of *Bacillus cereus* in wound infections

**DOI:** 10.1186/s12964-026-03235-x

**Published:** 2026-09-18

**Authors:** Naeem Ullah, Abdelbasset Yabrag, Anne Yska, Raswati Pant, Vignesh Ramnath, Toril Lindbäck, Laura M. Carroll, Manoj Puthia, Aftab Nadeem

**Affiliations:** 1 Department of Molecular Biology, Umeå University, Umeå, Sweden; 2 Umeå Centre for Microbial Research (UCMR), Umeå University, Umeå, Sweden; 3 Department of Clinical Microbiology, SciLifeLab, Umeå University, Umeå, Sweden; 4 Laboratory for Molecular Infection Medicine Sweden (MIMS), Umeå University, Umeå, Sweden; 5 Department of Paraclinical Sciences, Faculty of Veterinary Medicine, Norwegian University of Life Sciences, Ås, Norway; 6 Integrated Science Lab (IceLab), Umeå University, Umeå, Sweden; 7 Division of Dermatology and Venereology, Department of Clinical Sciences, Lund University, Lund, Sweden

**Keywords:** *Bacillus cereus*, NheABC, Wound infections, Spheroids, Organoids, Liposomes

## Abstract

Members of the *Bacillus cereus* group are Gram-positive, spore-forming, facultative anaerobic bacteria widely distributed in the environment. Pathogenic *B. cereus* group strains have been linked to a range of illnesses and infections, including severe non-gastrointestinal infections, such as wound infections. In the present study, we show that, among the pore-forming toxin (PFT)-producing bacterial strains tested, *B. cereus* group strain 75/95 induced pronounced membrane permeabilization in epithelial cells, prompting us to investigate the contribution of its tripartite NheABC toxin to host cell damage. NheABC exhibited potent cell membrane permeability in 2D epithelial cell cultures as well as in 3D spheroids and intestinal organoids, with prominent effects on the cell membrane and mitochondria. Functional analysis using liposome leakage assays demonstrated that cardiolipin (CL) and phosphatidylethanolamine (PE) promote NheABC-mediated membrane permeabilization, suggesting that membrane lipid composition contributes to NheABC activity and mitochondrial damage. Moreover, using erythrocytes and epithelial cells as model systems, we further demonstrated that the cytolytic activity of NheABC is pH-dependent and is markedly reduced under acidic conditions (pH 5.5). This pH dependence was independently confirmed using liposome leakage assays. Importantly, NheABC enhanced bacterial colonization and the local inflammatory response in a murine model of wound infection. Overall, our study highlights the critical role of mitochondria and pH in regulating NheABC-mediated cytolytic activity in mammalian cells, which may lead to the development of novel therapeutic strategies for managing *B. cereus* group-related gastrointestinal and non-gastrointestinal infections.

**Graphical Abstract:**

**Figure:**
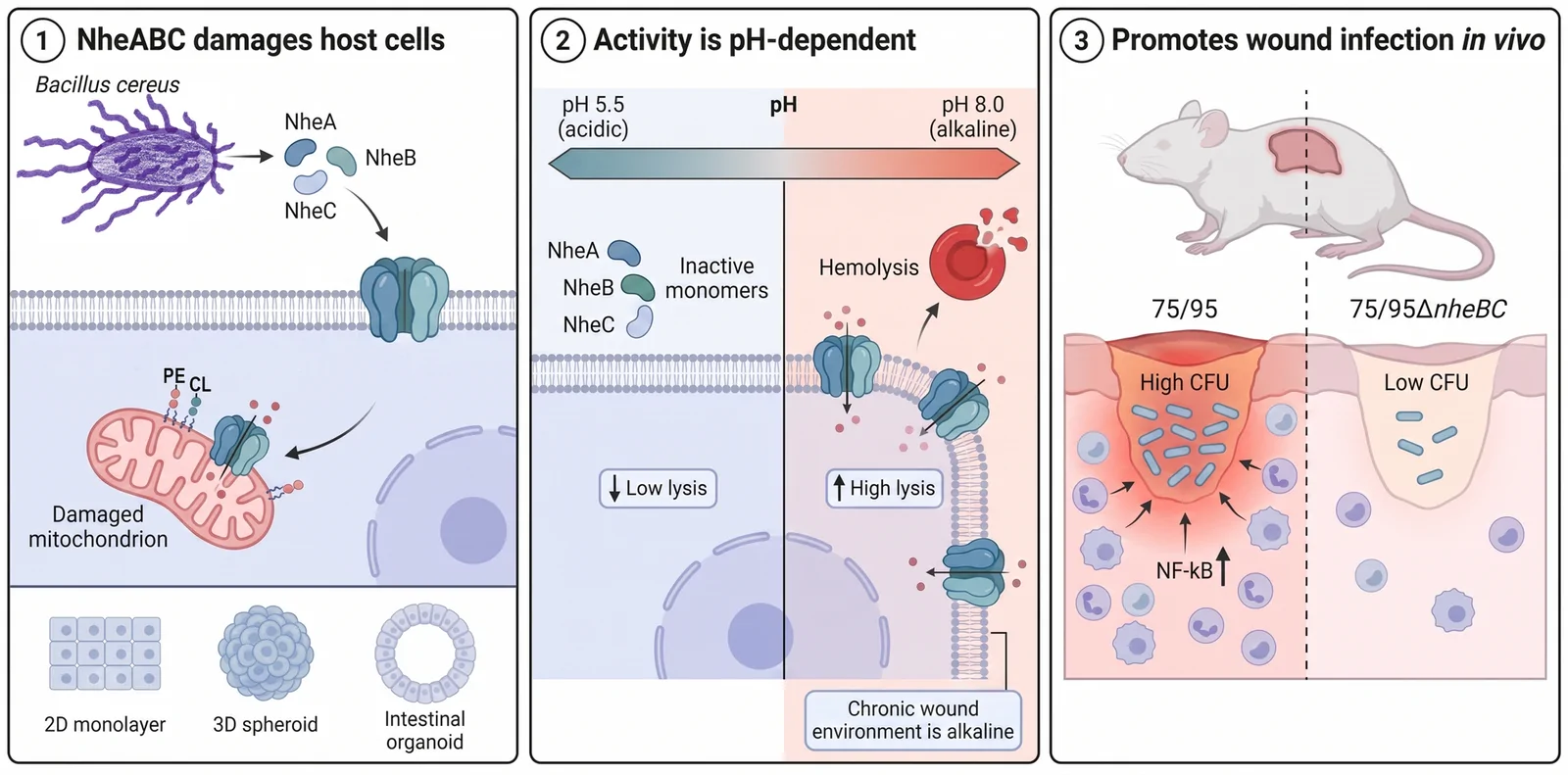

**Supplementary Information:**

The online version contains supplementary material available at https://doi.org/10.1186/s12964-026-03235-x.

## Introduction

Bacterial infections are a leading cause of morbidity and mortality worldwide. The incidence of lethal bacterial infections, including urinary tract infections, pneumonia, invasive bloodstream infections, skin and soft tissue infections, and wound infections, is rising worldwide. Pathogenic Gram-positive and Gram-negative bacteria produce pore-forming toxins (PFTs) as major virulence factors during mammalian host infections. These toxins play an important role in facilitating the colonization and systemic spread of pathogenic bacteria within the target host [1, 2]. Based on the secondary structure of the transmembrane regions responsible for their insertion into lipid membranes, PFTs can be classified into two major groups: α-PFTs (α-helical PFTs) and β-PFTs (β-barrel PFTs). These toxins are secreted as soluble, inactive monomers. Upon interaction with the target host lipid membranes, they undergo conformational changes that trigger oligomerization, ultimately forming the multimeric pores, required for mammalian cell lysis [3]. The α-PFTs comprises single-component cytotoxins, such as ClyA from *Escherichia coli* [4], as well as two-component toxins, including XaxAB from *Xenorhabdus nematophila*, XaxAB-like binary toxin from *Photorhabdus luminescens*, and the YaxAB from *Yersinia enterocolitica*. Additionally, this group includes three-component toxins, such as MakABE from *Vibrio cholerae*, AhlABC from *Aeromonas hydrophila*, SmhABC from *Serratia marcescens*, Hbl-BL_1_L_2_ and NheABC from the *Bacillus cereus* group [5–8].

Members of *B. cereus* group are Gram-positive, spore-forming bacteria widely distributed in various environments, including air, water, and soil. Within the *B. cereus* group, individual strains vary in terms of their pathogenic potential, and a range of illnesses and infections have been attributed to *B. cereus* group members, including foodborne gastrointestinal disease and non-gastrointestinal infections—such as skin, soft tissue, and wound infections [9–12]. Wound infections are often underestimated issues that can lead to chronic infections. Due to the presence of blood, interstitial fluid, and metabolic byproducts like ammonia, the pH of a chronic wound typically shifts to alkaline range, which may support the growth and colonization of bacterial pathogens, including pathogenic *B. cereus* group strains [10, 13].

While individual *B. cereus* group strains vary in terms of the risk that they pose to human health, virtually all *B. cereus* group strains possess homologs of non-hemolytic toxin-encoding NheABC [14–16]. NheABC is a tripartite pore-forming toxin encoded by the genes *nheA*,* nheB*, and *nheC*. All three components (NheA, NheB and NheC) in 10:10:1 molar ratio are required for the full cytolytic activity of the toxin [9, 11, 17]. The NheABC toxin was first isolated from the culture supernatant of *B. cereus* group strain NVH 00075/95, a *panC* Group III/Sequence Type 26 (ST26) strain [18], responsible for a food poisoning outbreak in Norway in 1995 [19]. This classification utilizes *panC* phylogenetic group assignment [15] and PubMLST’s seven-gene multi-locus sequence typing scheme for “*B. cereus*”. *B. cereus* group taxonomy is not standardized, but for simplicity, we will refer to NVH 00075/95 as “*B. cereus* (75/95)”.

For many years, mammalian cell cultures have served as invaluable tools for understanding the complex molecular and cellular mechanisms of bacterial infections. Most of these studies utilize two-dimensional (2D) cell cultures as model systems [20–22]. However, 2D cell culture models have clear limitations, as they do not reflect the complexity of native mammalian tissue physiology [23]. Although 2D cell cultures are inexpensive and highly reproducible, they lack many physiologically relevant features, such as cell-cell interactions, functional tight junctions, and the complex extracellular microenvironment necessary for regulating cell signaling [21]. Consequently, these limitations reduce the translational value of findings derived from 2D cell culture models. In contrast, while animal models offer a more comprehensive understanding of human pathophysiology and host-pathogen interactions. They present significant challenges, including high costs, time-consuming protocols, and substantial ethical concerns regarding animal welfare. To overcome these shortcomings, three-dimensional (3D) cell culture models, such as spheroids and organoids have gained increasing importance in the field of infection biology in recent years [23–27]. In 3D models, cells grow and interact in all three spatial dimensions, closely resembling in vivo microstructures. Moreover, certain cell lines grown as 3D spheroids, along with most organoids facilitates the development of cell-extracellular matrix, and cell-cell interactions. This results in tissue architectures that closely resemble in vivo conditions, making them optimal systems for studying bacterial infections [26–28].

In the present study, we report that among bacteria producing single-component, two-component, or three-component toxins, the tripartite NheABC toxin produced by a *B. cereus* (75/95) induced the maximum cell membrane permeability in infected or treated epithelial cells. Functional experiments using a liposome leakage assay suggested that NheABC primarily targets cardiolipin (CL) and phosphatidylethanolamine (PE) in the mitochondria of target epithelial cells. Similar to its effects in 2D monolayers, NheABC exhibited potent cell membrane permeability in 3D spheroids and intestinal organoids. Moreover, using erythrocytes and epithelial cells as model systems, we demonstrated that the biological activity of NheABC is pH-dependent, a finding further confirmed by a liposome leakage assays. Finally, NheABC enhanced the colonization of the *B. cereus* (75/95) and contributed to the activation of NF-κB in a non-gastrointestinal murine wound infection model.

## Results

### Bacterial pore-forming toxins induce cell membrane permeability in epithelial cells grown in 2D culture

**Fig. 1:**
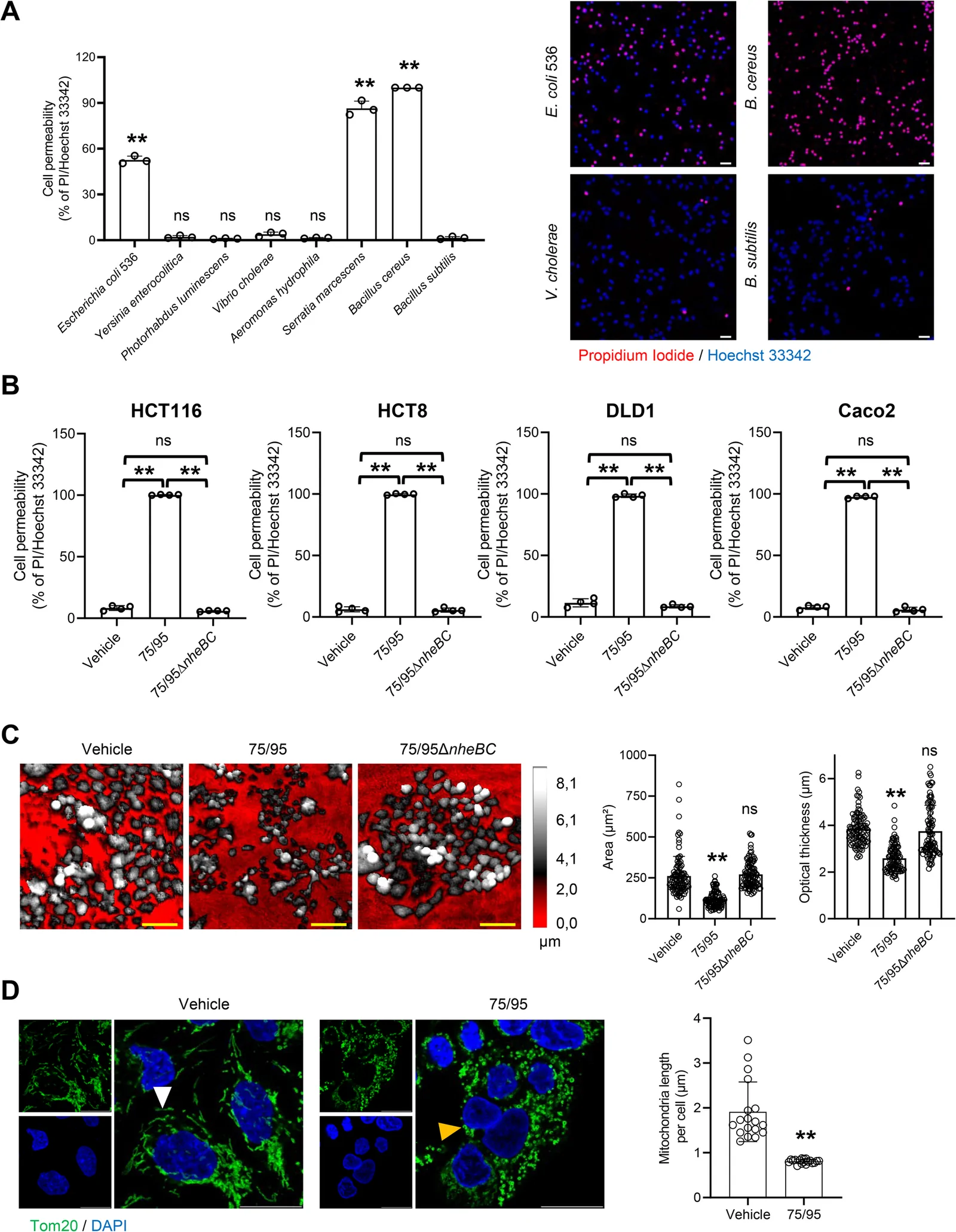
The *B. cereus* (75/95) NheABC toxin causes rounding of mitochondria and induces cell membrane permeability in epithelial cells. **A** HCT116 epithelial cells were infected with various bacterial species (MOI, 1:50) in complete DMEM media for 4 h at 37 °C, followed by staining with propidium iodide (red, staining dead cells), and Hoechst 33342 (blue, staining dead and alive cells). Images were acquired using the SparkCyto imaging system, followed by image segmentation using Cellpose. The total number of propidium iodide-positive dead cells was normalized against the Hoechst 33342-positive total number of cells. Scale bars = 20 μm. The bar chart shows the percentage of cell membrane permeability in response to various bacterial species. Data points represent replicates from independent experiments; bar graphs show mean ± s.d. Significance was determined from replicates using a one-way analysis of variance (ANOVA) with Sidak’s post-test against *B. subtilis*. **p < 0.01, ns = non-significant. **B** HCT116, HCT8, DLD1, and Caco-2 cells in the presence of propidium iodide and Hoechst 33342 were exposed to bacteria-free supernatant from the wild-type (75/95) or mutant (75/95Δ*nheBC*) strains of *B. cereus* for 4 h at 37 °C, following image acquisition with SparkCyto. Data points represent three replicates from independent experiments; bar graphs show mean ± s.d. Significance was determined from replicates using a one-way analysis of variance (ANOVA) with Sidak’s post-test. **p < 0.01. **C** HCT8 cells were exposed to bacteria-free supernatant from the wild-type (75/95) or mutant (75/95Δ*nheBC*) strains of *B. cereus* for 4 h at 37 °C, following image acquisition with HoloMonitor. The color gradients in the images, from red to white, indicate varying levels of cell thickness, with white representing the thickest cells. Scale bars = 50 μm. The bar chart to the right indicates changes in area and optical thickness. Data points in the bar chart represent individual HCT8 cells (n = 100) from two independent experiments; bar graphs show mean ± s.d. Significance was determined using a one-way analysis of variance (ANOVA) with Sidak’s post-test against the untreated cells. **p < 0.01. **D** HCT8 cells were exposed to vehicle (5% LB) or bacteria-free supernatant (5%) from the wild-type *B. cereus* (75/95) for 30 min. Mitochondria were detected with anti-Tom20 antibody (green), and nuclei were counterstained with DAPI (blue). White arrowheads indicate healthy tubular mitochondria, while orange arrowheads indicate round, fragmented mitochondria. Scale bars = 20 μm. The bar chart to the right in panel (**D**) shows a decrease in mitochondrial length in response to supernatant from wild-type *B. cereus*. Data points represent quantification of mitochondrial length from 18 random cells; bar graphs show mean ± s.d. Significance was determined using unpaired Student’s t-test. **p < 0.01

Numerous pathogenic bacterial species release single-component, two-component, or three-component PFTs. Upon interaction with membranes, these toxins undergo conformational changes, resulting in the formation of an oligomeric pores that leads to cell membrane permeabilization [1]. To address the role of PFTs-mediated epithelial cell membrane disruption, we exposed HCT116 cells either to various bacterial species at a multiplicity of infection of 50 (MOI, 1:50) or to bacteria-free supernatants for 4 h. Cells were subsequently stained with propidium iodide (to detect compromised cell membranes) and Hoechst 33342 (to stain all nuclei), and images were acquired using the SparkCyto imaging system. Data analysis demonstrated that the tripartite, NheABC-producing *B. cereus* (75/95) strain induced the highest level of cell membrane permeability in the infected epithelial cells. Similarly, *S. marcescens*, another tripartite PFT-producing bacterium, also induced increased epithelial cell membrane permeabilization. Importantly, in addition to the tripartite SmhABC, *S. marcescens* is known to produce ShlA—a β-PFT that may also contribute to epithelial cell membrane permeabilization [29]. We used *E. coli* 536, which produces the β-PFT, alpha-hemolysin as a positive control, while *B. subtilis*, which lacks PFTs, served as a negative control (Fig. 1A). The other tripartite (*A. hydrophila*, and *V. cholerae*) and bipartite PFT-producing bacteria (*Y. enterocolitica*, and *P. luminescens*) failed to induce cell membrane permeability in the infected epithelial cells (Fig. 1A). Consistent with the bacterial infection data, we observed maximum cell membrane permeabilization for HCT116 cells exposed to bacteria-free supernatants collected from the *B. cereus* (75/95) strain (Supplementary Fig. 1). These experiments therefore identify *B. cereus* (75/95) as a strong inducer of epithelial membrane permeabilization under the conditions used in this study and provide the rationale for further investigating the contribution of NheABC to this phenotype.

*B. cereus* (75/95) is known to produce the tripartite NheABC toxin. However, it also has the ability to produce a sphingomyelinase (SMase) that may contribute to cell membrane disruption [30]. To address the role of NheABC in *B. cereus* infection-mediated cell membrane permeabilization of epithelial cells, we exposed various epithelial cell lines to bacteria or bacteria free supernatant collected from the wild-type *B. cereus* (75/95), and the mutant lacking the NheBC components of the tripartite toxin (75/95Δ*nheBC*). Data analysis suggests that compared to the wild-type (75/95), the cell membrane permeabilization induced by the mutant (75/95Δ*nheBC*) strain was significantly attenuated (Fig. 1B and Supplementary Fig. 2A-B).

To determine if cell membrane permeabilization may lead to a loss of cell viability, we exposed HCT8 and DLD1 epithelial cells to bacteria-free supernatants collected from the wild-type (75/95) or mutant (75/95Δ*nheBC*) strain following MTS cell viability assay (Supplementary Fig. 3). Data analysis suggested that the loss of cell viability directly correlates with the increase in cell membrane permeabilization. Consistent with the cell membrane permeabilization and loss of cell viability data, we observed significant morphological changes in the HCT8 cells upon exposure to bacteria or bacteria-free supernatants (Fig. 1C and Supplementary Fig. 4A-B).

Mitochondrial integrity is essential for cellular homeostasis, and mitochondria have emerged as crucial battlegrounds in the interplay between host defenses and bacterial pathogens [31]. To determine whether NheABC targets the mitochondria of the epithelial cells, we exposed HCT8 cells to the bacteria-free supernatant collected from the wild-type (75/95) strain. This treatment induced mitochondrial rounding, as demonstrated by a reduction in mitochondrial length compared to the vehicle-treated cells (Fig. 1D and Supplementary Fig. 5). Together these results suggest that the NheABC toxin produced by the *B. cereus* (75/95) strain may directly target mitochondria of the epithelial cells.

### The NheABC tripartite toxin causes metabolic paralysis of target epithelial cells

**Fig. 2:**
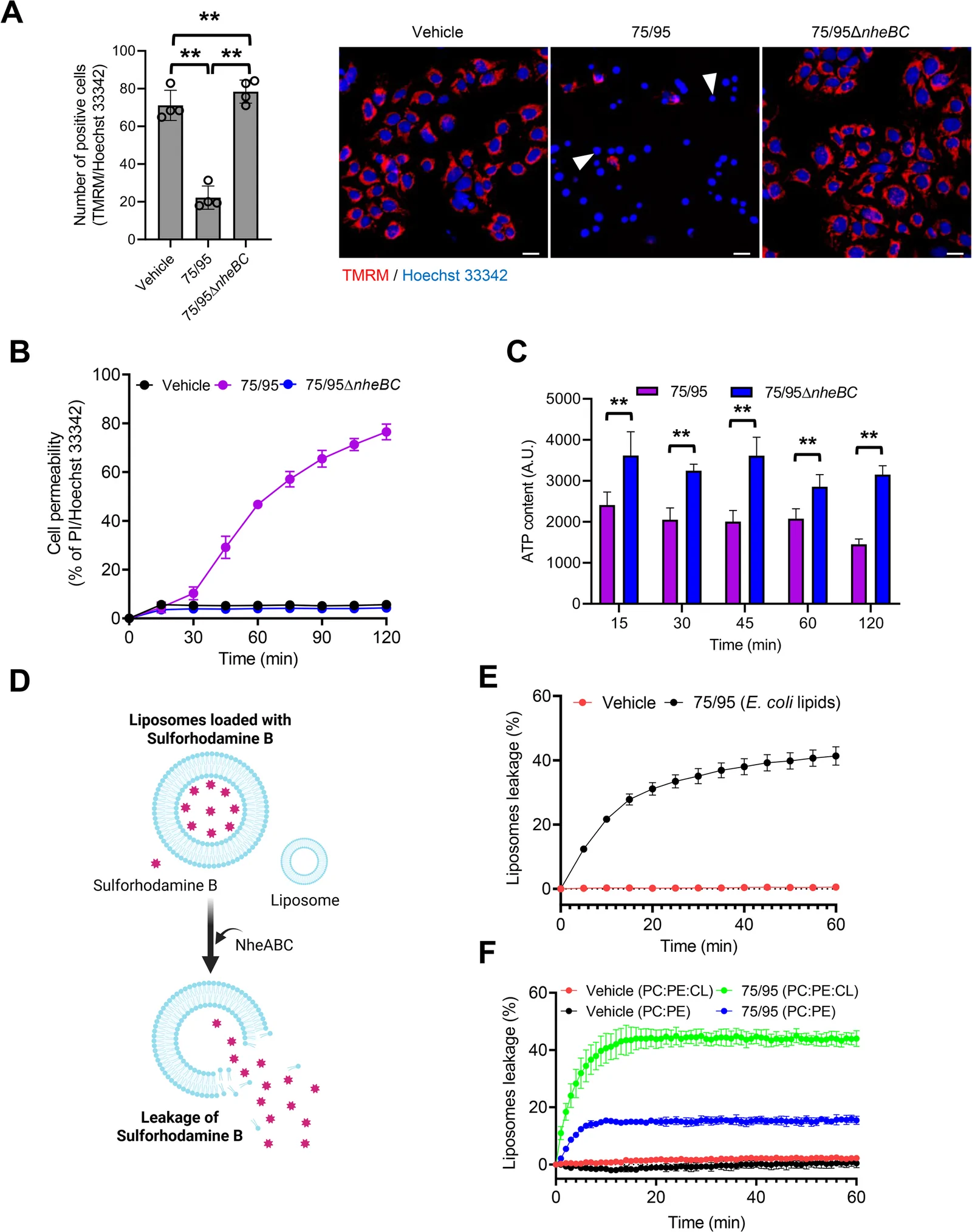
The tripartite toxin NheABC targets mitochondria and contributes to the metabolic paralysis of epithelial cells. **A** DLD1 cells were exposed to bacteria-free supernatant from the wild-type (75/95) or mutant (75/95Δ*nheBC*) strains of *B. cereus* for 60 min at 37 °C, followed by staining with TMRM (red), and Hoechst 33342 (blue). Arrowheads (white) indicate a loss of mitochondrial potential. Scale bars = 50 μm. Data points in the bar chart represent quantification of TMRM positive cells (n = 300–800) from four independent experiments; bar graphs show mean ± s.d. Significance was determined using a one-way analysis of variance (ANOVA) with Tukey’s multiple comparison post-test. **p < 0.01. **B** DLD1 cells pre-stained with Hoechst 33342 (2 µM) in RPMI-1640 media were exposed to bacteria-free supernatants (5%) collected from the wild-type *B. cereus* (75/95) and its isogenic mutant Δ*nheBC* (75/95Δ*nheBC*) strain. For vehicle control, cells were treated with LB (5%). Images were acquired every 15 min after treatment, and the number of propidium iodide positive cells was counted as dead cells. **C** DLD1 cells were exposed to bacteria-free supernatants (5%) collected from the wild-type *B. cereus* (75/95) strain or its isogenic mutant Δ*nheBC* (75/95Δ*nheBC*). Cells were lysed in a 96-well plate every 15 min, for a maximum of 120 min to calculate total cellular ATP. Data points represent three replicates from independent experiments; bar graphs show mean ± s.d. Significance was determined from replicates using unpaired Student’s t-test. **p < 0.01. **D** Schematic illustration of a liposome leakage assay performed with liposomes loaded with sulforhodamine B. The figure was created with https://www.biorender.com. **E** The line plot indicates time-dependent liposomes leakage induced by the wild-type *B. cereus* (75/95) supernatants, compared to the vehicle control in citrate buffer (120 mM, pH7.4). **F** The line plot indicates time-dependent kinetics of liposome leakage induced by wild-type *B. cereus* (75/95) supernatants, compared to the vehicle control (citrate buffer; 120 mM, pH7.4). The wild-type *B. cereus* (75/95) supernatants (5%) triggered increased leakage in liposomes prepared from synthetic lipids PC: PE: CL, compared to those composed of PC: PE. LB (5%) was used as a vehicle control in both panels **E** and **F**. Data are representative of 3 replicates

To investigate whether NheABC produced by the *B. cereus* (75/95) strain influences the mitochondrial integrity of the epithelial cells, DLD1 cells were infected with bacteria or treated with the bacteria-free supernatants collected from the *B. cereus* wild-type (75/95), or the mutant (75/95Δ*nheBC*) strain for 60 min, followed by staining with tetramethylrhodamine, methyl ester (TMRM), which accumulates in the active mitochondria of mammalian cells. Exposure of DLD1 cells to wild-type (75/95) strain or bacteria-free supernatant showed a significant decrease in the number of TMRM-positive cells compared to cells exposed to the mutant (75/95Δ*nheBC*) strain or the vehicle control (Fig. 2A and Supplementary Fig. 6A). To address whether the loss of mitochondrial potential leads to a decrease in total cellular ATP, we exposed DLD1 cells to bacteria-free supernatants isolated from both the wild-type (75/95) and the mutant (75/95Δ*nheBC*) strain. Exposure of DLD1 cells to the supernatant collected from the wild-type (75/95) strain caused a time-dependent increase in cell membrane permeabilization compared to cells exposed to supernatants collected from the mutant (75/95Δ*nheBC*) strain (Fig. 2B). Consistent with this NheABC-mediated loss of mitochondrial potential and increased cell membrane permeability, we observed a time-dependent decrease in the total cellular ATP content of DLD1 cells exposed to the wild-type (75/95) compared to the mutant (75/95Δ*nheBC*) strain (Fig. 2C). Importantly, we observed a rapid decrease in cellular ATP of ~ 50% within 15 min of treatment in response to wild-type (75/95) supernatants. In contrast, cell membrane permeability reached to ~ 50% at 60 min post-treatment, suggesting that among other cellular targets, mitochondria might be the major target for NheABC.

To address whether NheABC targets phospholipids found in the mitochondria of epithelial cells, we first prepared liposomes from *E. coli* lipid extracts encapsulated with sulforhodamine B and exposed them to the supernatant isolated from wild-type *B. cereus* (75/95). We observed a time-dependent increase in liposome leakage in response to the wild-type *B. cereus* (75/95) supernatant compared to the vehicle control (Fig. 2D-E). Bacterial membranes are composed of phospholipids such as phosphatidylglycerol (PG), phosphatidylethanolamine (PE) and cardiolipin (CL) [32]. Importantly, the mitochondria of epithelial cells are primarily composed of phosphatidylcholine (PC), PE, and CL [33]. We therefore, prepared liposomes consisting of PC: PE (70%:30%), or PC: PE: CL (40%:30%:30%). Exposure of these liposomes to the supernatant collected from wild-type *B. cereus* (75/95) resulted in a time-dependent increase in liposomal leakage (Fig. 2E-F). Importantly, we observed an increase in the leakage of liposomes prepared from PC: PE: CL, compared to those composed of PC: PE (Fig. 2F). To address whether this lipid-mediated leakage is specific to NheABC we exposed PC: PE: CL liposomes to supernatants collected from the *B. cereus* wild-type (75/95) or mutant (75/95Δ*nheBC*) strains. Data analysis indicated that these effects are specific to NheABC (Supplementary Fig. 6B). Together, these results suggest that the NheABC may target CL and PE in the mitochondria of epithelial cells.

### Components of the NheABC tripartite toxin accumulate in the mitochondria of target epithelial cells

**Fig. 3:**
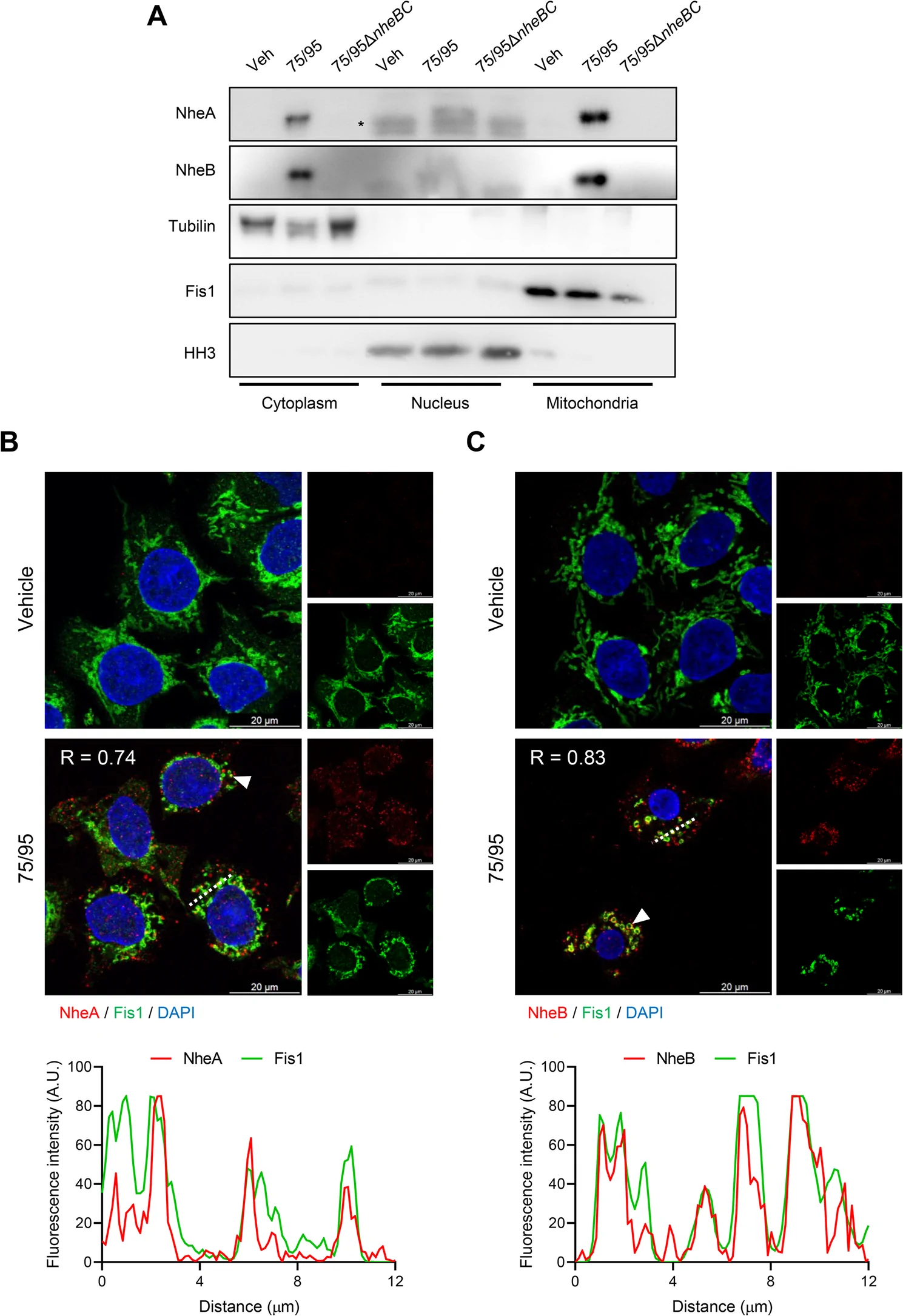
The components of NheABC tripartite toxin are present in the mitochondria of the epithelial cells. **A** Western blot analysis of NheA and NheB in various fractions of DLD1 cells. Cells were exposed to bacteria-free supernatants from the wild-type (75/95) or mutant (75/95Δ*nheBC*) *B. cereus* strains for 30 min at 37 °C, following cell fractionation and Western blot analysis. The purity of different cellular fractions was confirmed by probing the membrane for Tubulin (cytoplasm), Histone H3 (nuclear) and Fis1 (mitochondria). Data are representative of two or three independent experiments. The asterisk (*) indicates a non-specific band detected in the nuclear fraction of the cell. **B**-**C** Confocal microscopy of HCT8 cells exposed to the bacteria-free supernatant from the wild-type (75/95) *B. cereus* strain for 30 min at 37 °C. NheA and NheB were detected with anti-NheA or anti-NheB antibodies (red), whereas mitochondria were detected using anti-Fis1 antibodies (green), and nuclei were stained with DAPI (blue). Scale bars = 20 μm. Fluorescence intensity profiles along the dotted white line were used for calculating the Pearson correlation co-efficient

To address whether the components of NheABC accumulate in the mitochondria of target epithelial cells, we exposed DLD1 cells to bacteria-free supernatants collected from the wild-type (75/95) or its mutant (75/95Δ*nheBC*) strain, followed by cellular fractionation and Western blot analysis. Data analysis demonstrated that NheA and NheB were present in both the cytoplasmic and mitochondrial fractions of the cells exposed to the wild-type (75/95) supernatant. Importantly, neither NheA nor NheB was detected in the nuclear fraction (Fig. 3A and Supplementary Fig. 12). The mitochondrial localization of NheA and NheB was further confirmed by confocal microscopy, which revealed co-localization of NheA and NheB with the mitochondrial marker Fis1 (mitochondrial outer membrane fission 1) (Fig. 3B-C). Together, these results suggest that components of the NheABC toxin accumulate in the mitochondria of target epithelial cells.

### NheABC produced by *B. cereus* (75/95) causes cell membrane permeability in 3D spheroids and organoids

**Fig. 4:**
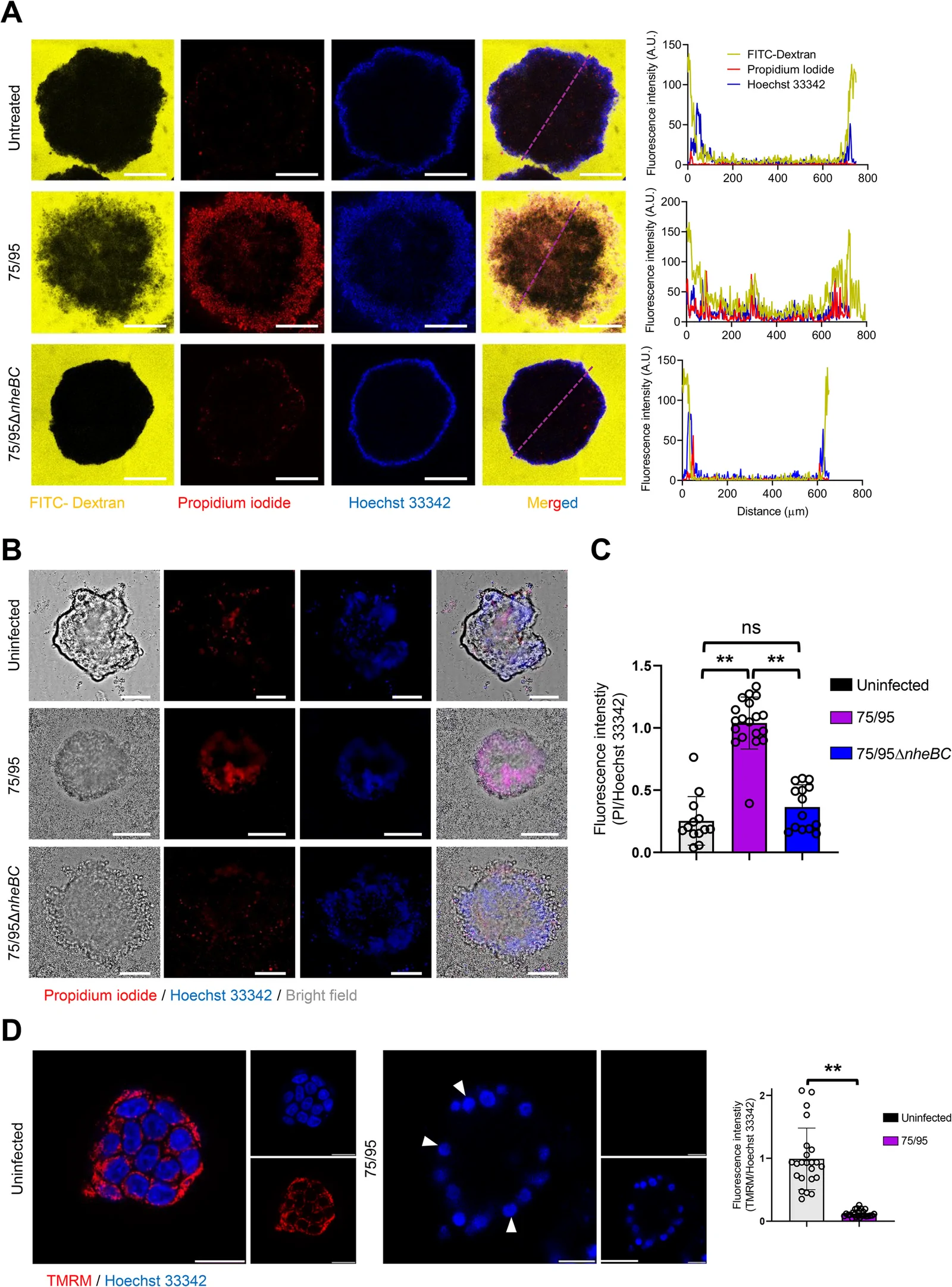
*Bacillus cereus* (75/95) targets the cell membrane and mitochondria of the infected intestinal organoid. **A** The DLD1 spheroids formed in a 96-well plate for 72 h were transferred to an 8-well glass bottom slide were exposed to bacteria-free supernatant from the wild-type (75/95) or mutant (75/95Δ*nheBC*) strains of *B. cereus* for 4 h at 37 °C, followed by staining with propidium iodide (red), Hoechst 33342 (blue), and FITC-Dextran (yellow). Scale bars = 200 μm. The line plot to the right indicates an increase in the number of propidium iodide positive cells and an increase in the penetration of FITC-Dextran into the lumen of the spheroids exposed to bacteria-free supernatant from wild-type (75/95), compared to the mutant (75/95Δ*nheBC*) supernatant treated or untreated spheroids. **B** Intestinal organoids were infected with the wild-type *B. cereus* (75/95), or its isogenic mutant (75/95Δ*nheBC*) strain for 4 h, followed by staining with propidium iodide (red), and Hoechst 33342 (blue). Scale bars = 50 μm. **C** The bar chart indicates an increase in the fluorescence intensity of propidium iodide in organoids infected with the wild-type *B. cereus* (75/95), compared to its isogenic mutant (75/95Δ*nheBC*) strain or uninfected organoids. Data points represent quantification of fluorescence from individual intestinal organoid (n = 13 to 19); bar graphs show mean ± s.d. Significance was determined using a one-way analysis of variance (ANOVA) with Sidak’s post-test against the uninfected organoids. **p < 0.01, ns = non-significant. **D** Intestinal organoids were infected with the wild-type *B. cereus* (75/95) for 4 h, followed by staining with TMRM (red), and Hoechst 33342 (blue). Arrowheads (white) indicate loss of mitochondrial potential. Scale bars = 20 μm. The bar chart to the right indicates a decrease in the fluorescence intensity of TMRM staining in organoids infected with the wild-type *B. cereus* (75/95), compared to the uninfected organoids. Data points represent quantification of cells in intestinal organoid (n = 23); bar graphs show mean ± s.d. Significance was determined using Student’s t-test. **p < 0.01

Two-dimensional (2D) cell culture models have been extensively used as tools to understand the cellular mechanisms involved in *B. cereus* group PFT-mediated mammalian cell membrane permeability and cell death [17, 34, 35]. However, these models have clear limitations as they lack complex cell-cell interactions and the extracellular microenvironment [21, 27]. Therefore, the use of three-dimensional (3D) cell culture models, including spheroids and organoids offers a physiologically relevant alternative to simplistic 2D cell culture methods. To investigate whether *B. cereus* group strains producing the NheABC toxin causes cell membrane permeability in 3D spheroids, we first developed spheroids from HCT116 or DLD1 cells and exposed them to either the wild-type *B. cereus* (75/95) or the mutant (75/95Δ*nheBC*) strain for 4 h, followed by propidium iodide and Hoechst 33342 staining (Supplementary Fig. 7A-B). Exposure to the wild-type *B. cereus* (75/95) strain caused disruption of the spheroid, as indicated by an almost complete loss of the spheroid core. Moreover, we observed a marked increase in the number of propidium iodide positive cells within spheroids exposed to the wild-type *B. cereus* (75/95) strain compared to those exposed to mutant (75/95Δ*nheBC*) strain or the untreated control (Supplementary Fig. 7B). To further understand the kinetics of NheABC-mediated cell membrane disruption in the HCT116 spheroids, we infected the spheroids with either the wild-type *B. cereus* (75/95) or mutant (75/95Δ*nheBC*) strain in the presence of propidium iodide, and images were acquired at 1 h interval for 5 h using the SparkCyto imaging system. Data analysis suggested a time-dependent increase in propidium iodide staining of the HCT116 spheroids exposed to the wild-type *B. cereus* (75/95) strain compared to the mutant (75/95Δ*nheBC*) strain (Supplementary Fig. 7C). To further confirm NheABC-mediated disruption of the 3D spheroid, we exposed DLD1 or HCT116 spheroids to bacteria or bacteria-free supernatants collected from the wild-type *B. cereus* (75/95) or mutant (75/95Δ*nheBC*) strain, followed by staining with propidium iodide, Hoechst 33342 and FITC-Dextran. Consistent with the SparkCyto live cell image analysis, confocal microscopy analysis suggested an increase in the number of propidium iodide-positive cells in spheroids exposed to wild-type *B. cereus* (75/95). Additionally, we also observed an increase in the FITC-Dextran permeability within the spheroids exposed to the bacteria or bacteria-free supernatants collected from the wild-type *B. cereus* (75/95) compared to the mutant (75/95Δ*nheBC*) strain (Fig. 4A and Supplementary Fig. 7D). Together, these results suggest that NheABC is the major virulence factor responsible for the disruption of HCT116 and DLD1 spheroids.

We next investigated the role of NheABC-mediated cell membrane permeability in the intestinal organoids, which are composed of various types of differentiated cells that self-assemble into a small, organ-like structures [36]. The intestinal organoids were infected with either the wild-type *B. cereus* (75/95) or the mutant (75/95Δ*nheBC*) strain for 4 h, followed by staining with propidium iodide and Hoechst 33342. Images were acquired using the SparkCyto imaging system. Data analysis indicated that organoids infected with the wild-type *B. cereus* (75/95) exhibited a significant increase in cell membrane permeability compared to the those infected with the mutant (75/95Δ*nheBC*) strain (Fig. 4B-C).

To determine whether *B. cereus* may causes mitochondrial dysfunction in intestinal organoids, similar to our observations in 2D monolayers, we infected the organoids with the wild-type *B. cereus* (75/95), followed by staining with TMRM and Hoechst 33342. Data analysis of images acquired with confocal microscopy revealed a marked decrease in TMRM staining in organoids infected with the wild-type *B. cereus* (75/95), compared to the uninfected organoids. Taken together, these data demonstrate that *B. cereus* (75/95) causes mitochondrial depolarization in the infected organoids (Fig. 4D). These results suggest that, consistent with the 2D cell culture data, the NheABC toxin targets the cell membrane and mitochondria of host cells in complex 3D cell cultures, such as spheroids and organoids.

### Targeting of the mammalian cell membrane by NheABC is pH-dependent

**Fig. 5:**
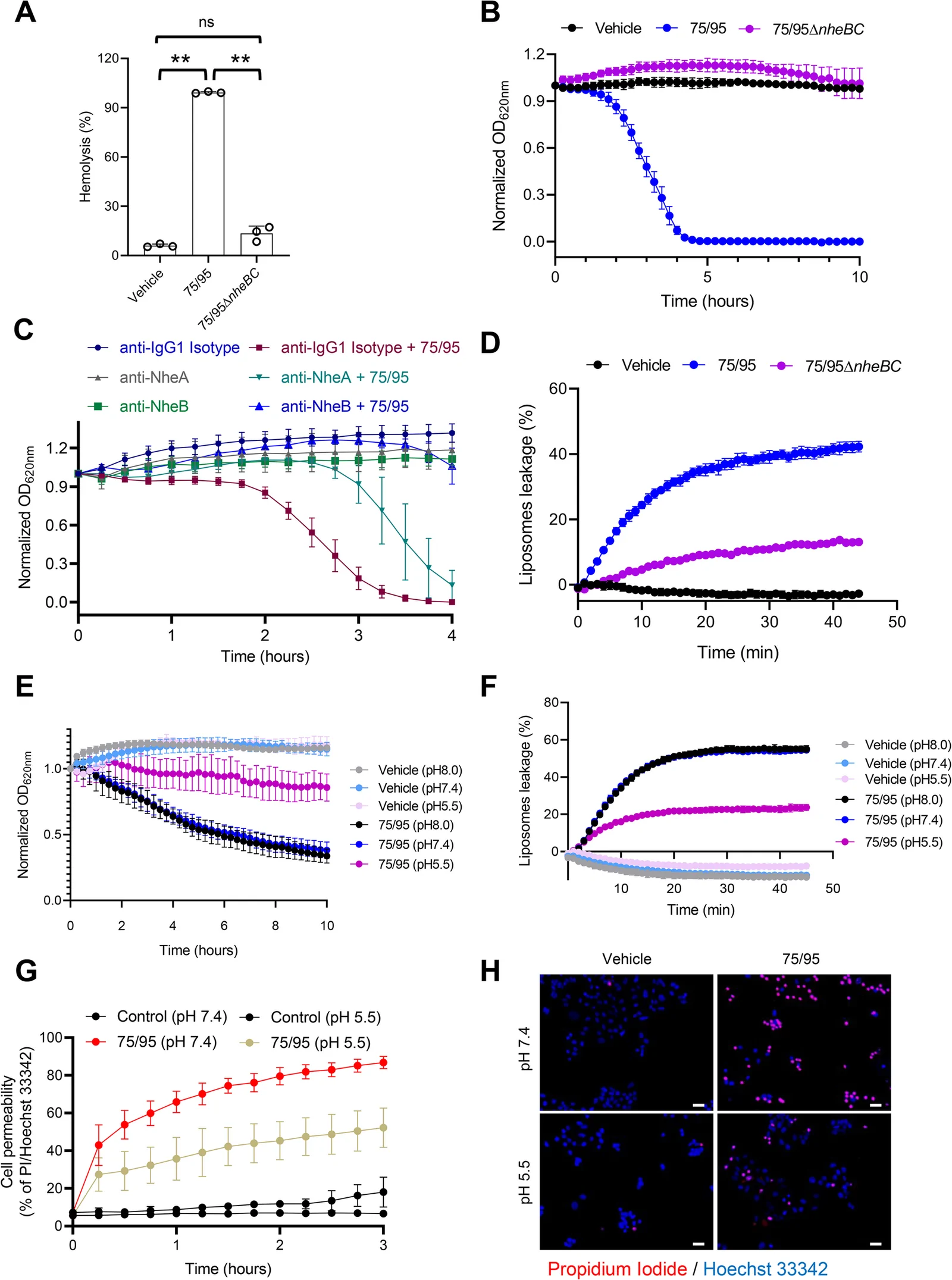
NheABC causes pH-dependent lysis of the target cell membrane. **A** Human erythrocytes were exposed to bacteria-free supernatant from the wild-type (75/95) or mutant (75/95Δ*nheBC*) strains of *B. cereus* for 4 h at 37 °C, followed by quantification of hemolysis at 415 nm. The data in bar chart show the percentage of hemolysis induced by the wild-type (75/95) or mutant (75/95Δ*nheBC*) treated erythrocytes. Data points represent three replicates from independent experiments; bar graphs show mean ± s.d. Significance was determined using a one-way analysis of variance (ANOVA) with Sidak’s post-test. **p < 0.01, ns = non-significant. **B** A turbidity assay was used to determine the kinetics of erythrocytes lysis by measuring its optical density every 10 min after the addition of bacteria-free supernatants (5% v/v) collected from the wild-type (75/95) or mutant (75/95 Δ*nheBC*) strain of *B. cereus* for 10 h at 37 °C. LB (5% v/v) was used as a vehicle control. **C** A turbidity assay was used to determine the kinetics of erythrocytes lysis by measuring its optical density every 10 min after the addition of bacteria-free supernatants (5% v/v) in the presence or absence of anti-NheA, anti-NheB or anti-IgG1 isotype control (1 µg/mL) for 4 h at 37 °C. **D** The line plot indicates time-dependent ECLE liposome leakage induced by the supernatants isolated from wild-type *B. cereus* (75/95) or mutant (75/95Δ*nheBC*) strain in citrate buffer (120 mM, pH7.4). **E** A turbidity assay was used to determine the kinetics of erythrocytes lysis by measuring the optical density of human erythrocytes every 10 min after the addition of the bacteria-free supernatants (5% v/v) collected from the wild-type *B. cereus* (75/95) for 10 h at 37 °C. Vehicle refers to LB (5% v/v), and citrate buffer (120 mM) of the indicated pH. **F** The line plot indicates time-dependent ECLE liposome leakage induced by the wild-type *B. cereus* (75/95) supernatant in citrate buffer (120 mM), at the indicated pH. Data in panels (**D**-**F**) are representative of two or more independent experiments. Data presented in the graphs is representative of three or more replicates. **G** HCT8 cells pre-stained with Hoechst 33342 (2 µM) in RPMI-1640 media of pH 7.4 or 5.5 were exposed to bacteria-free supernatants (5%) collected from the wild-type *B. cereus* (75/95). Images were acquired every 15 min after treatment, and the number of propidium iodide positive cells was counted as dead cells. Data in the line graph for panels (**B**-**G**) show mean ± s.d. from three replicates. **H** Representative images at a two hour time-point from the data presented in panel (**G**). Scale bars = 20 μm

Upon ingestion of contaminated food, *B. cereus* group strains capable of causing gastrointestinal illness colonize the small intestine of infected individuals [16]. In non-gastrointestinal wound infections, injuries exposed to soil or contaminated water may lead to *B. cereus* group strain exposure [37, 38]. Thus, the ability of pathogenic *B. cereus* group strains to adapt to diverse pH environments is crucial to their virulence against the mammalian host. The pH of the small intestine and colon can drop as low as 6.0 [39], while the pH of chronic wounds can range from 7.2 to 9.0 [13]. To investigate the role of pH in NheABC-mediated mammalian cell lysis, we first exposed human erythrocytes to bacteria-free supernatants collected from *B. cereus* wild-type (75/95) or mutant (75/95Δ*nheBC*) strain for 4 h. Consistent with the earlier studies, data analysis from the erythrocyte hemolysis assay suggested that the wild-type (75/95) *B. cereus* strain caused almost complete hemolysis, whereas the mutant (75/95Δ*nheBC*) strain remained inactive in this assay (Fig. 5A). We then investigated the kinetics of human erythrocyte cell lysis using a turbidity assay (Fig. 5B, and Supplementary Fig. 8A-B). Consistent with the hemolytic assay, we observed a concentration- and time-dependent decrease in erythrocyte turbidity ( which is indicative of hemolysis) in response to the supernatants collected from the wild-type *B. cereus* (75/95) strain, while supernatants from the mutant (75/95Δ*nheBC*) strain remained inactive (Fig. 5B and Supplementary Fig. 8A-B).

To determine whether the observed hemolytic effects are specific to NheABC, we performed an antibody neutralization experiment. Data analysis from the turbidity assay clearly indicated that monoclonal anti-NheA (1G4) antibodies partially neutralized the NheABC-mediated erythrocyte lysis, while monoclonal anti-NheB (1E11) antibodies almost completely blocked it (Fig. 5C). Moreover, we also performed genetic complementation of *nheBC* (75/95Δ*nheBC/*p*nheBC*) in the mutant (75/95Δ*nheBC*) strain. The expression of the complemented NheB component was confirmed by Western blot analysis (Supplementary Fig. 9A and Supplementary Fig. 12). Furthermore, we observed a time-dependent increase in erythrocyte lysis in response to the complemented strain (75/95Δ*nheBC/*p*nheBC*) compared to the vector control (75/95Δ*nheBC/*pVector) strain (Supplementary Fig. 9B).

To determine the role of mammalian cell lipids in NheABC-mediated mammalian cell lysis, we extracted lipids from epithelial cells (epithelial cell lipid extracts, ECLE) and loaded them with sulforhodamine B. We exposed the liposomes to bacteria-free supernatants collected from *B. cereus* (75/95) or the mutant (75/95Δ*nheBC*) strains. We observed a time-dependent increase in the release of sulforhodamine B in response to the wild-type (75/95) supernatant. Additionally, we observed minimal leakage of the liposomes in response to the mutant (75/95Δ*nheBC*) supernatants (Fig. 5D). While this effect could be explained due to the presence of SMase present in the mutant supernatant, it warrants further investigation.

To investigate the role of pH in NheABC-mediated erythrocyte lysis, we exposed erythrocytes to various pH conditions (ranging from pH 5.5 to 8.0) and quantified erythrocyte lysis using a turbidity assay. Data analysis indicated that erythrocyte lysis was maximum at pH 8.0 and was markedly reduced under acidic pH conditions (Fig. 5E). Consistent with this decrease in NheABC-mediated lysis of human erythrocytes, we also observed a reduction in the NheABC-mediated leakage of liposomes prepared from ECLE under acidic conditions (Fig. 5F). Additionally, the pH-dependent membrane damaging activity of NheABC was further confirmed in DLD1 and HCT8 epithelial cells (Fig. 5G-H and Supplementary Fig. 10A-B). Taken together, these data suggest that an acidic pH reduces NheABC-mediated mammalian cell membrane permeabilization.

### Diverse *B. cereus* group strains have been isolated from wounds in humans

**Fig. 6:**
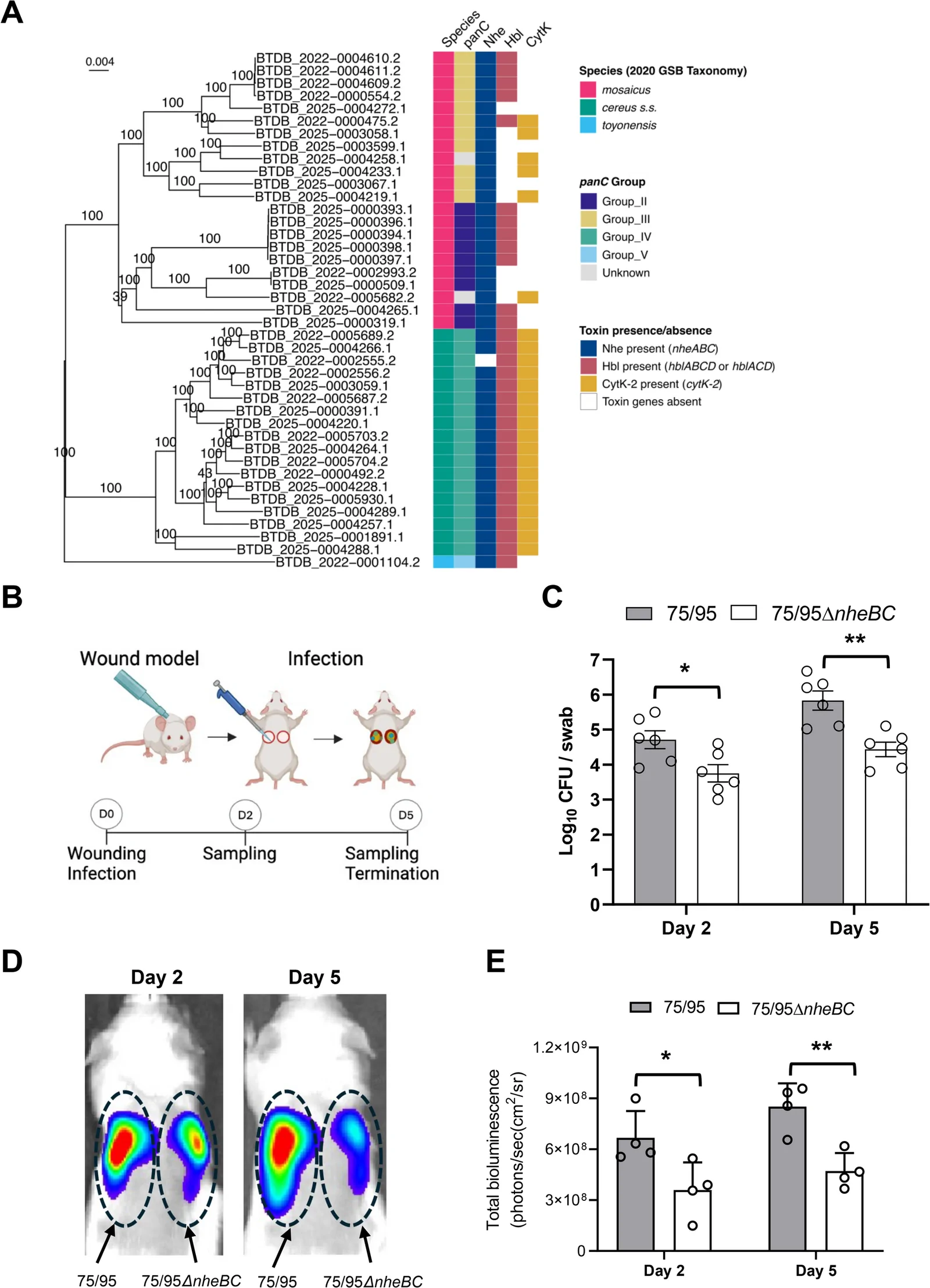
NheABC contributes to virulence of *B. cereus* group strains in wound infections. **A** Diverse *B. cereus* group strains have been isolated from wounds in humans. The distance-based tree (left) was constructed using Mashtree and displays 41 B. cereus group genomes, which were reportedly isolated from wounds/lesions in humans. The heatmap (right) denotes (from left to right): (i) *B. cereus* group genomospecies (obtained using BTyper3’s 2020 Genomospecies-Subspecies-Biovar [GSB] Taxonomy); (ii) panC Group (obtained using BTyper3); the presence and absence of genes encoding (iii) non-hemolytic toxin (Nhe; *nheABC*), (iv) hemolysin BL (Hbl; either *hblABCD *or *hblACD*), and (v) cytotoxin K variant 2 (CytK-2; *cytK-2*), all detected using BTyper3 and default thresholds (i.e., 70% and 80% amino acid identity and coverage, respectively). The Mashtree distance-based tree is rooted at the midpoint, with branch labels denoting bootstrap support percentages (out of 100 repetitions). Bootstrap support percentages on some internal nodes have been omitted for readability (see Supplementary Fig. 11 for the raw, unedited version of this figure). **B** Schematic illustration of the wound infection experiment. **C** A bar chart indicates recovery of wild-type *B. cereus* (75/95) or its isogenic mutant Δ*nheBC* (75/95Δ*nheBC*) 2- and 5-days post-infection from the wound of the infected mice. Data points in the bar chart represents CFU counts for bacteria collected from the individual mouse; bar graphs show mean ± s.d. Significance was determined using unpaired Student’s t-test. *p < 0.05, **p < 0.01. **D** Representative images of in vivo inflammation imaging by IVIS in NF-κB reporter mice. **E** A bar chart shows measured light intensity emitted from the reporter mice shown in (**D**). Data points in the bar chart represents data collected from individual mouse; bar graphs show mean ± s.d. Significance was determined using unpaired Student’s t-test. *p < 0.05, **p < 0.01

*B. cereus* group strains have been associated with a range of non-gastrointestinal infections, including traumatic wound infections [10, 37, 40, 41]. However, the role of NheABC in *B. cereus* wound infections is not well known. Using BTyperDB (a *B. cereus* group genomic database with manually curated metadata) [42], we identified 41 genomes of good-to-high quality, which were reportedly isolated from human wound infections (Table S2). Notably, these 41 genomes were diverse and spanned multiple species (Fig. 6A). Specifically, using *panC* Groups as a proxy for species [15], the wound-associated strains belonged to *panC* Groups IV (18 genomes, 43.9%), III (11 genomes, 26.8%), II (9 genomes, 22.0%), and V (1 genome; 2.4%); 2 genomes (4.9%) could not be assigned to a *panC* Group with confidence but were placed among genomes from *panC* Groups II and III using whole-genome-based methods (i.e., Mashtree and BTyperDB’s average nucleotide identity [ANI]-based methods; Fig. 6A and Supplementary Fig. 11).

BTyper3 (as implemented in BTyperDB) was used to identify genes encoding known *B. cereus* group toxins within each of the 41 wound-associated genomes (Table S2). Using default amino acid identity and coverage thresholds (i.e., 70% and 80%, respectively), none of the wound-associated genomes (0%) possessed the anthrax toxin-encoding *cya*, *lef*, and *pagA* genes, nor did any possess cereulide synthetase-encoding genes *cesABCD* (Table S2). As observed previously for the entirety of the *B. cereus* group [14, 16], nearly all genomes possessed Nhe-encoding *nheABC* (40/41, 97.6%; Fig. 6A). Interestingly, one *panC* Group IV genome (i.e., strain SJ-S28, isolated in Memphis, Tennessee, United States, from a human skin infection) [43] lacked all of *nheABC* at default amino acid identity and coverage thresholds (BTyperDB ID BTDB_2022-0002555.2, NCBI BioSample accession SAMN07351956; Fig. 6A); even when both thresholds were lowered to 0%, *nheABC* could not be detected (Table S3).

In addition to Nhe, genes encoding Hbl (*hblABCD* and *hblACD*) were detected in the majority (31, 75.6%) of the 41 wound-associated *B. cereus* group genomes (Fig. 6A). Specifically, all genomes from *panC* Groups IV and V possessed genes encoding Hbl, whereas Hbl-encoding genes were variably present within *panC* Group II and III genomes (similar to results observed previously for *panC* Groups II-V) [14]. Variant 2 of the gene encoding CytK (*cytK-2*) was additionally detected in a majority of the genomes queried here (24 of 41 wound-associated genomes, 58.5%; Fig. 6A). Like Hbl (and similar to results observed previously) [14], CytK-2-encoding *cytK-2* was present in all *panC* Group IV strains and variably present within *panC* Groups II and III; however, *cytK-2* could not be detected in the wound-associated *panC* Group V genome at default thresholds (Fig. 6A).

### NheABC contributes to successful colonization of *B. cereus* (75/95) in wound infections

To date, little is known about the role of NheABC in non-gastrointestinal wound infections. To determine the role of NheABC in this context, we utilized a mouse model of excisional wound and infected the wounds with either the wild-type *B. cereus* (75/95) or its mutant (75/95Δ*nheBC*) strain (Fig. 6B). The CFU counts from the infected wounds demonstrated a time-dependent increase in bacterial load in mice infected with the wild-type *B. cereus* (75/95) compared to those infected with the mutant (75/95Δ*nheBC*) strain. The wild-type *B. cereus* (75/95) showed an increase in colonization at both 2 days and 5 days post-infection, compared to the mutant (75/95Δ*nheBC*) strain (Fig. 6C).

We next explored whether this difference in colonization between wild-type and mutant strain translates into a differential virulence phenotype. We quantified local inflammatory response using NF-κB reporter mice. Consistent with the increased colonization, the wild-type (75/95) strain induced significantly stronger inflammatory response in the wound, which was attenuated in mice infected with the mutant (75/95Δ*nheBC*) strain (Fig. 6D-E). Taken together, these results suggest that, similar to its role in gastrointestinal infections, NheABC may play an important role in non-gastrointestinal wound infections.

## Discussion

Pathogenic bacteria produce PFTs that can disrupt host cell membranes and contribute to bacterial virulence. In the present study, we demonstrate that the tripartite NheABC toxin produced by *B. cereus* (75/95) induces pronounced membrane and mitochondrial damage in mammalian epithelial cells. These effects were observed not only in conventional 2D monolayer cultures but also in 3D spheroids and intestinal organoids. Using liposome leakage assays, we further identified phosphatidylethanolamine (PE) and cardiolipin (CL) as lipid components that promote NheABC-mediated membrane permeabilization. Moreover, using erythrocytes and epithelial cells as model systems, we demonstrated that the biological activity of NheABC is pH-dependent. This pH-dependent biological activity was further confirmed using liposome leakage assays. Moreover, our bioinformatic analysis confirmed that *B. cereus* group isolates from human wound infections had a high frequency of NheABC (97.6%). Consistent with these observations, NheABC enhanced local bacterial colonization and the inflammatory response in a murine excisional wound infection model. Collectively, these findings identify membrane lipid composition and environmental pH as important determinants of NheABC activity and support a role for this toxin in wound infections caused by *B. cereus* group strains.

Mitochondria play a central role in eukaryotic cell physiology and regulate multiple processes essential for cellular homeostasis, including energy metabolism and cell death [44]. Consequently, multiple pathogenic bacteria have evolved strategies to subvert mitochondrial functions to support their survival and dissemination [44]. Several bacterial effector molecules are known to target and remodel the mitochondrial network. These effectors include the vacuolating cytotoxin A (VacA) of *Helicobacter pylori* [45], the secreted porin B (PorB) of *Neisseria gonorrhoeae* [46], the secreted effector MitF from *Legionella pneumophila* [47], and the listeriolysin O (LLO) from *Listeria monocytogenes* [48]. Similar to these secreted bacterial effectors, NheABC causes mitochondrial damage in epithelial cells, leading to mitochondrial rounding. This disruption of mitochondrial network induces mitochondrial membrane depolarization, as demonstrated by a reduction in TMRM staining. Importantly, our results from the kinetic experiments suggest that NheABC causes reduction in cellular ATP levels at 15-minute time point, while the cell membrane permeability is initiated at 30 min time point. The temporal separation between these events suggests that mitochondrial dysfunction may precede overt disruption of the plasma membrane and may therefore contribute to the early cellular response to NheABC. However, these observations do not establish whether mitochondrial dysfunction results from direct NheABC-mediated membrane permeabilization or from secondary cellular responses to toxin exposure, and this distinction will require further investigation.

Mitochondrial outer membrane permeabilization (MOMP) is a critical event in the induction of apoptosis in response to various stimuli [49]. Previous studies have shown that the NheABC toxin induces apoptosis in Vero epithelial cells [35]. Typically, in response to PFT-induced apoptosis, BAX is translocated to the outer mitochondrial membrane [50]. This process triggers the release of pro-apoptotic molecules, such as cytochrome *c*, from the mitochondria into the cytosol, activating the caspase cascade and apoptotic cell death [51]. During apoptosis mitochondria undergo drastic reorganization, including fragmentation (indicated by mitochondrial rounding), cristae remodeling, loss of mitochondrial membrane potential, changes in lipid composition, and MOMP [51]. In this context, CL becomes enriched at the mitochondrial membrane during apoptosis [52], suggesting a possible link between lipid composition and susceptibility of mitochondria to NheABC. An important finding of the current study is the presence of NheABC components within the mitochondria of target epithelial cells, coupled with its ability to target CL in liposomes prepared from synthetic lipids. This suggests that the localization of NheABC may induce pore formation in the mitochondria, potentially leading to rapid loss of cellular ATP and subsequent plasma membrane disruption. Given that cell membrane permeabilization and organelle stress are frequently associated with MOMP during apoptosis or necrosis [51], our ongoing work focuses on dissecting the cellular pathways underlying NheABC-induced mitochondrial damage. However, the precise molecular mechanisms governing this rapid ATP reduction CL targeting in mitochondria require further investigation. To explore the molecular determinants of these cellular effects, our biochemical liposome leakage assays indicated that NheABC causes a time-dependent leakage of liposomes prepared from *E. coli* polar lipids. Experiments using synthetic liposomes with defined lipid compositions further showed that the CL and PE promote maximal NheABC-mediated membrane leakage. CL is particularly abundant in mitochondrial membranes, whereas PE is an important structural phospholipid present in both mitochondrial and other cellular membranes. Together with the observed mitochondrial localization of NheABC components and the rapid mitochondrial dysfunction detected in epithelial cells, these findings suggest that the lipid composition of mitochondrial membranes may contributes to their susceptibility to NheABC. Nevertheless, the liposome leakage experiments measure membrane permeabilization, not direct lipid binding. Thus, additional biochemical and biophysical studies will therefore be required to determine whether individual NheABC components directly recognize CL, PE, or particular combinations of these lipids.

In this study, we initially compared the ability of several PFT-producing bacterial species to induce epithelial membrane permeabilization under identical conditions. Among the strains tested, *B. cereus* (75/95) produced the strongest membrane-permeabilizing phenotype. Importantly, however, this experiment represents a comparison of the overall cytotoxic phenotypes of the bacterial strains under the conditions used and should not be interpreted as a direct comparison of the intrinsic pore-forming activities of their respective PFTs. Expression, secretion, and abundance of individual toxins were not normalized across the different bacterial species and are likely to vary considerably according to bacterial growth and infection conditions. Indeed, some PFTs, such as the bipartite YaxAB toxin, are poorly expressed or undetectable under standard laboratory growth conditions [53]. Therefore, the absence of detectable membrane permeabilization by some of the bacterial strains does not indicate that their respective PFTs lack cytolytic activity. Rather, the initial comparative experiment served as a phenotypic screen that identified *B. cereus* (75/95) for subsequent investigation of the contribution of NheABC to epithelial cell damage.

During the last decade, 3D cell culture has rapidly advanced our understanding of tumor and infection biology, showing promising outcomes [27]. The 3D spheroids and organoid models closely mimic animal and human tissues, thereby reducing the reliance on animal models. In the current study, we utilized 3D spheroids prepared from HCT116 or DLD1 monolayers and intestinal organoids as a model systems to investigate NheABC-mediated targeting of mammalian cell membranes and mitochondria. We sought to determine if spheroids and organoids could be used as alternatives to 2D cell culture models to understand the cellular mechanisms involved in NheABC-mediated cellular responses in complex cellular systems that closely mimic in vivo conditions. Our results suggest that similar to our 2D cell culture findings, NheABC targets mammalian cell membranes and mitochondria in complex organoid cell cultures. Moreover, we were able to translate these findings to an in vivo murine wound infection model, where we observed NheABC-mediated enhanced colonization and inflammatory response of *B. cereus* (75/95) in the infected wounds. These results suggest that NheABC may play a critical role in *B. cereus* (75/95) invasion into deep tissues, which warrants further investigation.

In previous studies, several PFTs have been reported to exhibit enhanced interaction with lipid membranes under acidic pH conditions. These include the MakA cytotoxin (a component of the MakABE cytotoxin) from *V. cholerae* [6, 54, 55], , colicin A from *E. coli* [56], listeriolysin O (LLO) from *L. monocytogenes* [57] vacuolating cytotoxin A (VacA) from *H. pylori* [58], and perfringolysin O (PFO) from *Clostridium perfringens* [59]. Most of these PFTs undergo a pre-pore to pore transition under low pH conditions, thereby activating their cytolytic activities. Moreover, the effect of pH on the pore-forming ability of two *B. thuringiensis* toxins, Cry1Ac and Cry1C have been previously reported [60]. Cry1Ac is highly effective at alkaline pH, while Cry1C is active in an acidic to neutral pH environment [60]. The putative pH-sensing mechanism in NheABC remains unclear, but it likely relates to the protonation of specific amino acids, such as histidine residues, at low pH [54]. Importantly, it has been reported previously that in addition to α-PFTs such as MakA, a member of β-PFTs LLO is activated by acidic pH (< 6). At neutral pH, both MakA and LLO exhibit very little cytotoxic activity, but their activity is substantially increased at low pH (5.5). In contrast, the cell membrane damaging activity of NheABC is substantially decreased at low pH (5.5), while maximum cell toxicity was observed at pH 7.4 and above. A plausible explanation for this pH-dependent decrease in NheABC activity could be that an acidic pH may inhibit the association of the NheABC tripartite components with lipid membranes. However, this warrants further investigation.

To our knowledge, the influence of pH on the membrane-permeabilizing activity of major *B. cereus* group virulence factor, the tripartite NheABC toxin, has not been previously investigated. Our cellular and biochemical data demonstrate that the cytolytic activity of NheABC is highest at a slightly alkaline pH, whereas a drop toward acidic pH levels significantly decreases its cytolytic activity. These observations suggest that NheABC may act as a major virulence factor in bacterial infection sites where the pH is slightly alkaline, such as traumatic wound infections. By combining advanced in vitro cellular models with in vivo validation, we provide evidence that NheABC not only targets mammalian cell membranes and mitochondria but also enhances bacterial colonization and the local inflammatory response in a murine model of wound infection. Collectively, these findings further support a model in which the local microenvironment may potentiate toxin-mediated tissue damage and bacterial persistence, highlighting the need for direct pH measurements in infected wounds in future studies. Overall, our results suggest that targeting the pH-mediated activity of NheABC may lead to the development of novel therapeutics for treating both gastrointestinal and non-gastrointestinal infections caused by *B. cereus* group strains.

## Materials and methods

### Antibodies

Anti-Fis1 (#10956-1-AP, WB = 1:1000, IF = 1:100) antibody was purchased from Proteintech. Anti-Histone H3 (#06-755, WB = 1:3000) and anti-β-Tubulin (#T8328, WB = 1:5000) antibodies were purchased from Sigma-Aldrich. Anti-Tom20 (#612278, IF = 1:100) antibody was purchased from BD Biosciences. The previously characterized monoclonal antibodies anti-NheA [61] (#1G4, WB = 1:1000, IF = 1:300), and anti-NheB [35, 62] (#1E11, WB = 1:1000, IF = 1:300) were also used in the current study. Goat anti-rabbit-HRP (#AS09602, WB: 1:5000) secondary antibodies were purchased from Agrisera, while Rabbit anti-mouse-HRP (#P0260, WB: 1:5000) secondary antibodies were purchased from Dako. Alexa Fluor 488/555 conjugated secondary antibodies for immunofluorescence were purchased from Thermo Fisher.

### Growth conditions for bacterial strains

All bacterial strains used in this study are listed in Table S1. All the bacterial strains were grown on Luria/Lysogeny agar (LA) plates and incubated overnight at 37 °C, except for *Photorhabdus luminescens*, and *Yersinia enterocolitica*, which were grown at 26 °C. To prepare bacteria-free supernatants for mammalian cell treatment, the bacterial strains were grown overnight in Lysogeny broth (LB) medium. The bacteria were subsequently removed by centrifugation (5000 × g for 10 min), and collected supernatants were passed through a 0.22 μm filter.

### Construction of complementing mutant 75/95Δ*nheBC*::pHT304-*nheBC*

For *trans*-complementation of the Δ*nheBC* mutant, a transcriptional fusion was constructed by linking the *nhe* promoter region (500 bp upstream of the *nhe* operon) to *nheBC*. The fusion was generated by sequence- and ligation-independent cloning (SLIC)-PCR using DreamTaq DNA Polymerase (Thermo Fisher Scientific). The resulting SLIC-AD amplicon was subsequently cloned into pCR2.1-TOPO (Thermo Fisher Scientific). The SLIC-A and SLIC-D primers included a *PstI* restriction enzyme site (CTGCAG), which facilitated transfer of the AD fragment to the low-copy-number shuttle vector pHT304 [63]. pHT304-*nheBC* was passed through One Shot™ INV110 *E. coli* (ThermoFisher Scientific) to achieve unmethylated DNA to enhance the transformation efficiency in *B. cereus*. The unmethylated plasmid was introduced into the mutant (75/95Δ*nheBC*) strain by electroporation [64]. As a negative control, the expression vector pHT304 without an insert was introduced into the mutant (75/95Δ*nheBC*) strain. The insert was amplified by PCR and sequenced (Eurofins). The sequence revealed a change from Ser^348^ to Pro in NheB and changes from Glu^275^ to Lys and Thr^286^ to Pro in NheC, in addition to five silent mutations. The following PCR primers were use.

SLIC-A: CTG*CTGCAG*AAAGGGAATGAAAATACTTC

SLIC-B: GTTACTTATTTTTACATTCTAATTGAATTCGATA

SLIC-C: TCAATTAGAATGTAAAAATAAGTAACGATATAG

SLIC-D: ATAC*CTGCAG*ATAAGAAGGTTGGTACTCT

*PstI* restriction enzyme sites are highlighted in italic font.

### Cell cultures

HCT116, HCT8, Caco-2 and DLD1 cell lines were maintained in RPMI-1640 media (Sigma-Aldrich) supplemented with 10% fetal bovine serum (FBS), 1% penicillin/streptomycin, and non-essential amino acids at 37 °C with 5% CO_2_.

### Ethics statement

All animal experiments were performed according to Swedish Animal Welfare Act SFS 1988:534 and were approved by the Animal Ethics Committee of Malmö/Lund, Sweden. The study was conducted in accordance with the local legislation and institutional requirements.

### Cell permeability and cell viability

The bacterial strains were grown on Luria Agar (LA) plates at 37 °C overnight, as specified above. The HCT116, and HCT8 cell lines were grown in RPMI-1640 medium supplemented with 10% fetal bovine serum (FBS) and incubated at 37 °C overnight in an incubator with continuous flow of 5% CO_2_. Cells were seeded in a 96-well plate (1 × 10^4^ cells/well), or 24-well plate (5 × 10^4^ cells/well). The following day, cells were washed with PBS and exposed to bacteria (MOI, 1:50) or bacteria-free supernatant for 4 hours in DMEM or RPMI-1640 media.

Cell membrane permeability was measured by staining the cells with a mixture of propidium iodide (1 µg/mL) and Hoechst 33342 (2 µM) for 30 min. Images were acquired using the SparkCyto imaging system (Tecan). For accurate and efficient segmentation of cells Cellpose [65] or StarDist [66] were used. The segmented images were further analyzed with ImageJ- FIJI distribution [67].

Cell viability was determined using an MTS (3-(4,5-dimethylthiazol-2-yl)-5-(3-carboxymethoxyphenyl)-2-(4-sulfophenyl)-2H-tetrazolium) cell viability assay (Promega, G3582). Briefly, HCT8 or DLD1 cells were seeded in a 96-well plate (1 × 10⁴ cells/well) and exposed to bacteria-free supernatants (5%) collected from the wild-type *B. cereus* (75/95) or the mutant (75/95Δ*nheBC*) strain for 4 h at 37 °C. At the end of the treatment MTS was added to each well, and absorbance was measured at 490 nm using a Spark microplate reader (Tecan).

### Kinetics of epithelial cell membrane permeability and estimation of total cellular ATP

DLD1 cells (1 × 10^4^ cells/well) were seeded in a 96-well plate and incubated overnight at 37 °C and 5% CO_2_. The following day, DLD1 cells were incubated with a mixture of propidium iodide (1 µg/mL) and Hoechst 33342 (2 µM) for 30 min and exposed to bacteria-free supernatants (5%) collected from the wild-type *B. cereus* (75/95) or its mutant (75/95 Δ*nheBC*) strain. Images were acquired on the SparkCyto imaging system every 15 min for a maximum duration of 120 min at 37 °C with a continuous flow of 5% CO_2_. The acquired images were analyzed as discussed above.

For the measurement of total cellular ATP, DLD1 cells (1 × 10^4^ cells/well) were seeded in a 96-well plate and incubated at 37 °C overnight with 5% CO_2_. The following day, DLD1 cells were exposed to bacteria-free supernatants (5%) collected from the wild-type *B. cereus* (75/95) or its mutant (75/95 Δ*nheBC*) strain. Cells were lysed in a 96-well plate every 15 min for a maximum duration of 120 min to determine total cellular ATP levels. Cellular ATP levels were quantified using the ATPLite kit (PerkinElmer, #6016943).

### Measurement of mitochondrial potential

DLD1 cells were grown in a 96-well plate (1 × 10^4^ cells/well) in RPMI-1640 medium supplemented with 10% fetal bovine serum (FBS) and incubated at 37 °C overnight in an incubator with continuous flow of 5% CO_2_. The following day, cells were washed with PBS and exposed to bacteria (MOI, 1:50) or bacteria-free supernatant collected from wild-type *B. cereus* (75/95) or its mutant (75/95Δ*nheBC*) strain for 60 min in a serum free DMEM media. At the end of the experiment cells were stained with mitochondrial potential dye TMRM (100 nM) and Hoechst 33342 (2 µM) for 30 min, followed by image acquisition using the SparkCyto imaging system (Tecan), and analysis using StarDist, Cellpose and ImageJ- FIJI distribution [67].

### Preparation and treatment of 3D spheroids

To develop spheroids, HCT116 and DLD1 (ATCC) cells were seeded in a cell repellent 96-well plate (BIOFLOAT 96-well cell culture plate [#F202003]) for 72 h using Cancer Stem Premium (#20141-500) supplemented with non-essential amino acids. The spheroids were exposed to bacteria (10^4^ bacteria) or bacteria-free supernatants collected from the wild-type *B. cereus* (75/95) or the mutant (75/95Δ*nheBC*) strain for 4 hours at 37 °C. Cell permeability was measured by staining the cells with a mixture of propidium iodide (1 µg/mL) and Hoechst 33342 (2 µM) for 30 min, followed by image acquisition using the SparkCyto imaging system (Tecan) or Confocal microscopy. The acquired images were analyzed using ImageJ FIJI distribution [67].

### Preparation of intestinal organoids culture and bacterial infection

Colon intestinal organoids were purchased from EMD Millipore, an affiliate of Merck KGaA (14-881CR) SCC310. Matrigel matrix basement membrane (Corning, #356234) was used for the preparation of the dome. Human basal medium (IntestiCult OGM, #100–0150) and organoids supplement (#100–0151) were purchased from STEMCELL Technologies and used as medium for the maintenance of organoid culture. Gentle cell dissociation reagent (#100–0485, STEMCELL Technologies organoids) was used to dissociate organoids from the dome. Organoids (*n* = 20–30) were infected with the wild-type *B. cereus* (75/95) or the mutant (75/95Δ*nheBC*) strain (10^4^ bacteria) in Cancer Stem media supplemented with 1% Matrigel for 4 hours at 37 °C. Cell permeability was measured by staining the intestinal organoids with a mixture of PI (1 µg/mL) and Hoechst 33342 (2 µM) for 30 min, followed by image acquisition using the SparkCyto imaging system (Tecan) or Confocal microscopy. Images were analyzed using ImageJ FIJI distribution [67].

### Holographic microscopy

Holographic microscopy was performed using the HoloMonitor^®^ M4 (Phase Holographic Imaging AB) equipped with a motorized stage to investigate the changes in epithelial cell morphology. HCT8 cells were cultured overnight in RPMI-1640 medium in a 96-well (1 × 10^4^) or 24-well (5 × 10^4^ cells/well) plates. The following day, cells were exposed to bacteria (MOI: 50) or bacteria-free supernatant collected from the wild-type *B. cereus* (75/95) or the mutant (75/95Δ*nheBC*) strain for 4 hours at 37 °C, after which images were acquired using the HoloMonitor^®^ M4. This system generates label-free images reconstructed into three-dimensional holograms. Quantitative measurements, such as average cell thickness and area, were extracted using Hstudio™ software [68].

### Confocal microscopy

To investigate mitochondrial morphology, HCT8 cells (1 × 10⁴ cells/well) were seeded overnight in an 18-well, glass-bottom chamber slide (µ-Slide, ibidi) and incubated at 37 °C. The following day, the cells were exposed for 30 min to either a vehicle control (5% LB) or bacteria-free supernatant (5%) collected from the wild-type *B. cereus* (75/95) strain. Subsequently, the cells were fixed with paraformaldehyde and permeabilized with 0.25% Triton X-100 in PBS. The samples were then incubated overnight at 4 °C with anti-Tom20 (BD Biosciences, #612278; 1:100 dilution in 5% FCS-PBS) or anti-Fis1 (Proteintech, # 10956-1-AP; 1:100 dilution in 5% FCS-PBS) antibodies and washed three times with PBS. After washing, the cells were incubated with Alexa488-conjugated secondary antibodies (Thermo Fisher, 1:200) for 1 h at room temperature. Nuclei were counterstained with DAPI (Sigma Aldrich, 1 µg/mL 5% FCS-PBS) for 5 min and washed three times with PBS. Images were acquired using a Leica SP8 inverted confocal system (Leica Microsystems).

For co-localization experiments, HCT8 cells after treatment as discussed above were fixed and permeabilized, following incubation with anti-NheA (1 µg/mL in 5% FCS-PBS) or anti-NheB (1 µg/mL in 5% FCS-PBS) in combination with anti-Fis1 (Proteintech, # 10956-1-AP; 1:100 dilution in 5% FCS-PBS) antibodies overnight at 4 °C. After three PBS washes, cells were incubated with Alexa488, and Alexa568-conjugated secondary antibodies (Thermo Fisher, 1:200) for 1 h at room temperature. Nuclei were counterstained with DAPI (Sigma Aldrich, 1 µg/mL 5% FCS-PBS) for 5 min and washed three times with PBS. Images were acquired using a Leica SP8 inverted confocal system (Leica Microsystems). Fluorescence intensity profiles were generated using the plot profile function in ImageJ, and image processing was performed using the ImageJ–FIJI distribution [67].

For the preparation of 3D culture cells for confocal microscopy, HCT116 or DLD1 spheroids and intestinal organoids were exposed to bacteria (10^4^ bacteria) or bacteria-free supernatant (5%) collected from the wild-type *B. cereus* (75/95) or the mutant (75/95Δ*nheBC*) strain for 4 hours in an 8-well chamber slide with a coverslip bottom (µ-Slide, ibidi). HCT116 or DLD1 spheroids were stained with a mixture of propidium iodide (1 µg/mL), Hoechst 33342 (2 µM), and FITC-Dextran 70 kDa (0.2 mg/mL) for 30 min.

Organoids were infected with the wild-type *B. cereus* (75/95) (10^4^ bacteria) in Cancer Stem media supplemented with 1% Matrigel for 4 hours at 37 °C. Loss of mitochondrial potential was investigated by staining the organoids with TMRM (100 nM), and Hoechst 33342 (2 µM) for 30 min, followed by image acquisition using the confocal microscopy.

Fluorescence intensity profiles were generated using the plot profile function in ImageJ, and image processing was performed using the ImageJ–FIJI distribution [67].

### Western blot analysis

For cell fractionation experiment, DLD1 colorectal cancer cells (3 × 10⁶) were treated with bacteria-free supernatant from the wild-type *B. cereus* (75/95) or the mutant (75/95Δ*nheBC*) strain for 30 min at 37 °C. Cells were then rinsed with PBS and lysed using the Qproteome cell compartment kit (#37502, Qiagen) according to the manufacturer’s instructions. The mitochondrial fraction of the cell was isolated using mitochondria isolation kit (ab110170, Abcam) according to manufacturer’s instructions. Briefly after the isolation of each fraction, protein estimation was performed using BCA assay, according to manufacturer’s instructions. Cell lysates were mixed with 4× sample buffer, and based on the protein estimation from BCA assay equal amount of proteins were loaded to the SDS-PAGE.

The Western blot samples for *B. cereus* (75/95Δ*nheBC*) mutant strain complemented with pHT304:nheBC (75/95Δ*nheBC*/p*nheBC*) and a pHT304 vector control (75/95Δ*nheBC*/pVector) were grown for 6 h in the presence of erythromycin (5 µg/mL), and the supernatants were collected as mentioned above. Proteins in the supernatant were precipitated with 50% (w/v) trichloroacetic acid (TCA). Samples were centrifuged for 20 min at 15,000 × g. The pelleted TCA-precipitated proteins were resuspended in 1× SDS sample buffer.

For both experiments, protein lysates were boiled for 5 min and separated by SDS-PAGE. The proteins were then transferred on to PVDF membranes, which were blocked in skim milk (5% prepared in 0.1% PBST) for 1 h at room temperature, following incubation of the membranes with the primary antibodies in blocking buffer (5% skimmed milk prepared in 0.1% PBST) overnight at 4 °C. Following antibodies were used for the detection of specific proteins, anti-NheA (0.1 µg/mL in PBST) and anti-NheB (0.1 µg/mL in PBST), anti-Tubulin (1:3000 dilution, cytoplasm marker), anti-Histone H3 (1:3000 dilution in PBST, nuclear marker), and anti-Fis1 (1:1000 dilution in PBST, mitochondrial marker). After washing with PBST (0.1%), membranes were incubated with appropriate HRP-conjugated secondary antibodies in blocking buffer for 1 h at room temperature. Protein bands were detected using chemiluminescence reagent (Bio-Rad) and images were acquired using the ImageQuant LAS 4000 instrument.

### Human erythrocytes lysis assay

For kinetic measurements of erythrocyte lysis, human erythrocytes (1% v/v) were exposed to bacteria-free supernatants in a phosphate buffered saline (PBS) at indicated concentrations. Erythrocytes were treated with 5% (v/v) of bacteria-free supernatant from either wild-type *B. cereus* (75/95) or the mutant (75/95Δ*nheBC*) strain. Erythrocytes treated with 5% (v/v) LB was used as a negative control, while erythrocytes treated with TritonX-100 (0.025% v/v) were used as a positive control.

For antibody neutralization experiments, bacteria-free supernatants were pre-incubated with the corresponding anti-NheA (#1G4) or anti-NheB (#1E11) antibodies at a final concentration of 1 µg/mL, which was then added to the erythrocytes. The IgG1 isotype (#66360-1-PBS) antibodies were used as a control for these experiments. For erythrocytes exposed to the bacteria-free supernatant collected from the complemented pHT304:nheBC (75/95Δ*nheBC*/p*nheBC*) strain, the pHT304 vector control (75/95Δ*nheBC*/pVector) served as a control.

Turbidity of the erythrocytes was monitored every 10 min by measuring optical density at 620 nm using Spark microplate reader (Tecan) with intermittent orbital shaking at 37 °C.

The kinetics of erythrocytes cell lysis under various pH conditions (pH 8.0, pH 7.4, and pH 5.5) in response to bacteria-free supernatant from wild-type *B. cereus* (75/95) was performed in various pH-adjusted citrate buffers (120 mM).

The percentage (%) of erythrocyte cell lysis was calculated using the following formula:

The diluted erythrocytes were added into the 96-well microplate wells, and turbidity of the samples was measured as F_0_. After the addition of 5 µl of bacteria-free supernatant, the turbidity was continuously measured as *F*_*tn*_ every 15 min for 10 h. The lowest turbidity after the addition of TritonX-100 was measured as *F*_*100*_. The erythrocytes lysis is defined in two steps as, Step1: F_1_ = (F_0_ or *F*_*tn*_–*F*_*100*_), and Step2: F_tn_/F_1_. Each erythrocytes lysis assay was performed in three to four technical replicates. The experiment was repeated at least three times.

### Lipid extraction and preparation of liposomes for leakage assays

Epithelial cell lipids were extracted from three 75 cm² confluent flasks of DLD1 colon cancer cells using the Folch method [69]. Briefly, cells were harvested, pelleted, and resuspended in a 2:1 chloroform-methanol mixture to extract lipids. The organic phase was collected, and the lipid extract was dried under a gentle nitrogen stream to form a thin lipid film, yielding approximately 5 mg of dried lipids.

Liposomes were prepared from total lipid extracts of *E. coli* obtained commercially from Avanti Polar Lipids (#100500 C). For the preparation of liposomes from synthetic lipids, 1-palmitoyl-2-oleoyl-glycero-3-phosphocholine (PC; #850457), 1,2-dioleoyl-sn-glycero-3-phosphoethanolamine (PE; 850725), and cardiolipin (CL; #710337) were mixed in a molar (%) ratio of 40:30:30 or PC and PE were mixed in a molar (%) ratio of 70:30. The lipids were dissolved in chloroform and dried under a gentle nitrogen stream to form a thin lipid film.

To investigate leakage of liposomes in response to NheABC, sulforhodamine B leakage assay was performed. Sulforhodamine B was dissolved at 50 mM in a sodium citrate buffer (120 mM). Lipid films (1 mg) were rehydrated with 1 mL sulforhodamine B solution. The hydrated lipid suspensions were extruded 11 times through a 100 nm polycarbonate membrane using an Avanti Mini-Extruder (Avanti Polar Lipids) at 40 °C to form unilamellar liposomes. Unbound sulforhodamine B was removed from the loaded liposomes using Sephadex G-50 chromatography.

To perform liposome leakage assay in response to bacteria-free supernatants collected from wild-type *B. cereus* (75/95) or its isogenic mutant (75/95Δ*nheBC)* strain, sulforhodamine B encapsulated liposomes were diluted 20-fold with citrate buffer (120 mM, pH 7.4). The kinetics of liposomes leakage was investigated using Spark plate reader (Tecan).

The kinetics of ECLE leakage assay under various pH conditions (pH 8.0, pH 7.4, and pH 5.5) in response to bacteria-free supernatant from wild-type *B. cereus* (75/95) was performed. The fluorescence of sulforhodamine B acid was measured in a Spark plate reader with excitation of 540 nm and emission filter of 620 nm. The 20 times diluted liposomes were added into the 96-well microplate wells, and the fluorescence emission was recorded as *F*_*0*_, 5 µl of bacteria -free supernatant was added into each well, and the fluorescence emission was continuously measured as *F*_*tn*_ every 1 min for 45 to 60 min. At the end of the experiment, 5 µl of 0.25% TritonX-100 was added to each well for maximum leakage of the liposomes. Addition of 5 µl of LB was used as a negative control. The highest fluorescence emission after the addition of TritonX-100 was measured as *F*_*100*_. The percentage of liposome leakage is defined by the following formula, [(Ftn - F0) / F100] × 100. Each liposome leakage assay was performed in three to four technical replicates. The experiment was repeated at least three times.

### Acquisition of human wound-associated *Bacillus cereus* group genomes

Human wound-associated *B. cereus* group genomes and their associated metadata were acquired from BTyperDB [42]. Briefly, all genomes meeting the following criteria were downloaded from BTyperDB (version 2025 November 30): (i) the strain was isolated from a human (`Source_1 == “Human”`); (ii) the strain was responsible for an “Other” type of illness (i.e., not foodborne and not anthrax; `Human_Illness == “Other”`) or an unknown type of illness (`Human_Illness == “Unknown”`); (iii) the strain’s metadata contained one or more of the following key words, in any field (case insensitive): `wound`, `lesion`, `skin infection`; (iv) the genome was not classified by BTyperDB as “low quality” (`Genome_Quality != “Low_Quality"`); (v) the genome did not belong to *B. anthracis* (i.e., *B. mosaicus* subsp. *anthracis*, using the 2020 Genomospecies-Subspecies-Biovar [GSB] taxonomy [70] assigned via BTyperDB’s implementation of BTyper3 [14]; to ensure strains from cutaneous anthrax cases were removed, `BTyper3_subspecies != anthracis`). The metadata for genomes that resulted from this search was manually inspected to confirm that each strain was isolated from a human wound. Overall, this search resulted in 41 human wound-associated genomes, which were used in subsequent steps (Table S2).

### Tree construction and visualization

Mashtree v1.4.5 [71] was used to construct a distance-based tree using the 41 human wound-associated *B. cereus* group genomes as input (see section “Acquisition of human wound-associated *Bacillus cereus* group genomes” above) and the following parameters: (i) the `mashtree_bootstrap.pl` command; (ii) 100 bootstrap repetitions (`--reps 100`); (iii) the `--mindepth` parameter set to 0 (to prioritize tree accuracy by ignoring very unique *k*-mers that are more likely read errors; `--mindepth 0`); (iv) a genome size of 5,619,812 bp (i.e., the median size of the 41 human wound-associated genomes; `--genomesize 5619812`); (v) 8 CPUs (`--numcpus 8`).

The resulting tree was plotted in R v4.4.0. Specifically, the Newick file produced by Mashtree was loaded into R using the `read.tree` function from ape v5.8-1 [72] and rooted at the midpoint using the `midpoint.root` function from phytools v2.4-4 [73]. Metadata from BTyperDB was loaded into R using the `read.delim` function (parameters: `header = T, sep = “\t”, stringsAsFactors = F, check.names = F`). The midpoint-rooted tree was plotted using the `ggtree` function from ggtree v3.12.0 [74]. The `gheatmap` function from ggtree was used to display the following metadata from BTyperDB in a heatmap next to the tree: (i) species (per BTyper3’s 2020 GSB Taxonomy); (ii) *panC* Group (per BTyper3); (iii) presence/absence of genes encoding Nhe (*nheABC*), Hbl (*hblABCD* or *hblACD*), and CytK (*cytK-1* or *cytK-2*; detected using BTyperDB’s implementation of BTyper3, default settings).

### Toxin gene detection in an Nhe-negative genome

According to BTyperDB, one genome (strain SJ-S28, BTyperDB ID BTDB_2022-0002555.2, NCBI BioSample accession SAMN07351956) lacked Nhe-encoding *nheABC* (a nearly ubiquitous toxin within the *B. cereus* group) [14, 16]. To confirm the absence of *nheABC*, the SJ-S28 genome was queried for toxin genes separately, using BTyper3 v3.4.0 [14] and amino acid identity and coverage thresholds lowered to 0 (`--virulence_identity 0 --virulence_coverage 0`). The resulting tblastn table was manually inspected to confirm that *nheABC* were absent (Table S3).

### Bacterial culture preparation for wound infection

Both wild-type *B. cereus* (75/95) and its mutant (75/95Δ*nheBC*) strains were used for wound infection experiments. A single bacterial colony was inoculated in a tube with 5 mL of Todd Hewitt Broth (THB) and incubated overnight at 37 °C in a shaking incubator. To refresh the culture the next day, 100 µL of the overnight culture was inoculated into a tube containing 5 mL of THB. The tube was then incubated at 37 °C in a shaking incubator until the optical density (OD) reached 0.4. Cultures were then centrifuged at 5,600 rpm for 10 min. The bacterial pellet was washed in 5 mL Tris buffer (10 mM, pH 7.4) and centrifuged again. The resulting pellet was then diluted in Tris buffer to a density of 10⁸ CFU/mL and used to infect wounds.

### Mouse model of wound infection

To study wound infection, a mouse model of excisional wound was used. BALB/c mice (8–10 weeks old male; Janvier Labs, France) were anesthetized by intraperitoneal injection of ketamine (60 mg/kg) and xylazine (10 mg/kg). The dorsal skin was shaved with clippers, and a depilatory cream was applied to remove any remaining hair. The skin was then disinfected with 10% povidone-iodine solution followed by wiping with a 70% alcohol swab.

A sterile 4 mm biopsy punch was used to create markings for two wounds—one on the left and one on the right side of the midline—with at least 5–6 mm distance between the two. Circular skin pieces were then excised with scissors. Wounds were covered with sterile gauze pieces. Thirty minutes later, mice were given 1 ml of prewarmed saline subcutaneously. For analgesia, buprenorphine (0.1 mg/kg mixed in saline) was administered.

Wounds were infected with 10 µL of bacterial suspension (10^8^ CFU/mL). The wounds were then covered with a polyurethane dressing (Mepilex Transfer; Mölnlycke, Sweden), and a transparent adhesive film dressing (Tegaderm; 3 M) was applied.

Under isoflurane anesthesia, observations were performed, and wound dressings were changed every other day. The experiment was terminated on day 5. During observation, sterile cotton swab samples were collected from wounds for further microbiological analysis. Swab bacterial CFU analysis was done as described before [75].

Male BALB/c tg (NFkB-RE-Luc)-Xen reporter mice (Taconic Biosciences), 10–12 weeks old, were used to study the inflammatory effects of wild-type (75/95) or its mutant (75/95Δ*nheBC*) strain. Similar bacterial infection protocol as discussed above was used. For IVIS (In Vivo Imaging System) imaging, the dorsum of the mouse was shaved carefully and cleaned. Mice were immediately transferred to individually ventilated cages. Bioimaging with the IVIS spectrum was used for the longitudinal determination of NF-κB activation. Fifteen minutes before IVIS imaging at Day 2 or Day 5 post-infection, mice were intraperitoneally injected with 100 µL of D-luciferin (PerkinElmer, 150 mg/kg body weight). Bioluminescence from the mouse was detected and quantified using Living Image 4.0 Software (PerkinElmer).

### Statistical analysis

Data are shown as mean ± s.d. Statistical significance was determined by one-way ANOVA, Student’s t-test or as otherwise stated in the corresponding figure legends. Statistical significance is indicated as *p* ≤ 0.01 (**) or *p* ≤ 0.05 (*). Graphs were produced and statistical analysis were performed using GraphPad Prism software.

## Supplementary Information

Supplementary Material 1.

Supplementary Material 2.

Supplementary Material 3

Supplementary Material 4

## Data Availability

No datasets were generated or analysed during the current study.
