# Supplementary material for: NheABC is a pH-dependent cytotoxin that targets mitochondria and contributes to the virulence of Bacillus cereus in wound infections
Source: Cell Commun Signal. 2026 Sep 18;24:500. doi: 10.1186/s12964-026-03235-x (PMC13587333; doi:10.1186/s12964-026-03235-x)
Supplement: Supplementary file 1 [file 12964_2026_3235_MOESM1_ESM.docx]

**Supplementary Information**

**NheABC is a pH-dependent cytotoxin that targets mitochondria and contributes to the virulence of *Bacillus cereus* in wound infections**

Naeem Ullah^1,2#^, Abdelbasset Yabrag^1,2#^, Anne Yska^1,2^, Raswati Pant^1,2^, Vignesh Ramnath^2,3,4^, Toril Lindbäck^5^, Laura M. Carroll^2,3,4,6^, Manoj Puthia^7^, Aftab Nadeem^1,2^

^1^Department of Molecular Biology, Umeå University, Umeå, Sweden

^2^Umeå Centre for Microbial Research (UCMR), Umeå University, Umeå, Sweden

^3^Department of Clinical Microbiology, SciLifeLab, Umeå University, Umeå, Sweden

^4^Laboratory for Molecular Infection Medicine Sweden (MIMS), Umeå University, Umeå, Sweden

^5^Department of Paraclinical Sciences, Faculty of Veterinary Medicine, Norwegian University of Life Sciences, Ås, Norway

^6^Integrated Science Lab (IceLab), Umeå University, Umeå, Sweden

^7^Division of Dermatology and Venereology, Department of Clinical Sciences, Lund University, Lund, Sweden

#These authors contributed equally to this work

**Corresponding author:** [aftab.nadeem@umu.se](mailto:aftab.nadeem@umu.se)

**Supplementary Figure 1:** **Cell membrane permeability in response to bacteria-free supernatants**

HCT116 epithelial cells were exposed to bacteria-free supernatants for 4 hours at 37°C, followed by staining with propidium iodide, and Hoechst 33342. Images were acquired using the SparkCyto imaging system, and image segmentation was performed using StarDist. The total number of propidium iodide-positive cells was normalized against the total number of Hoechst 33342-positive cells. The data in bar chart indicates the percentage of cell membrane permeability induced by various bacterial species. Data points represent replicates from independent experiments; bar graphs show mean ± s.d. Significance was determined from replicates using a one-way analysis of variance (ANOVA) with Dunnett’s post-test against *B. subtilis*. **p<0.01, *p≤0.05, ns = non-significant.

**Supplementary Figure 2:** **NheABC contributes to epithelial cell permeabilization during B. cereus infection**

**(A)** The bar graph indicates cell membrane permeability in HCT116 cells infected with the wild-type (75/95) or the mutant (75/95Δ*nheBC*) *B. cereus* strains for 4 hours at 37°C. Data points represent three replicates from independent experiments; bar graphs show mean ± s.d. Significance was determined from replicates using a one-way analysis of variance (ANOVA) with Sidak’s post-test. **p<0.01. Representative fluorescence images (right panel) show propidium iodide (red) and Hoechst 33342 (blue) positive cells. Scale bars = 20 µm. **(B)** The bar graph indicates cell membrane permeability in HCT8 cells infected with the wild-type (75/95) or mutant (75/95Δ*nheBC*) *B. cereus* strains for 4 hour at 37°C. Data points represent three replicates from independent experiments; bar graphs show mean ± s.d. Significance was determined from replicates using a one-way analysis of variance (ANOVA) with Sidak’s post-test. **p<0.01.

**Supplementary Figure 3:** **NheABC contributes to cell death in epithelial cells**

The bar graph indicate loss of cell viability in HCT8 and DLD1 cells exposed to bacteria-free supernatant from the wild-type (75/95) and the mutant (75/95Δ*nheBC*) *B. cereus* strains for 4 hours at 37°C. Data points represent four replicates from independent experiments; bar graphs show mean ± s.d. Significance was determined from replicates using a one-way analysis of variance (ANOVA) with Sidak’s post-test. **p<0.01, ns = non-significant.

**Supplementary Figure 4:** **NheABC contributes to changes in epithelial cell morphology**

**(A)** HCT8 cells infected with the wild-type (75/95) *B. cereus* or mutant (75/95Δ*nheBC*) strains at an MOI, 1:50 for 4 hours at 37°C were imaged with quantitative holographic microscopy. The color gradient in the images, from red to white, indicate varying levels of cell thickness, with white representing the thickest regions. Scale bars = 100 µm. **(B)** The bar graph indicates alterations in cellular area and optical thickness under various treatment conditions. Data points in the histogram represent individual HCT8 cells (n=96) from two independent experiments; bar graphs show mean ± s.d. Significance was determined from replicates using a one-way analysis of variance (ANOVA) with Sidak’s post-test against the uninfected cells. **p<0.01, *p≤0.05.

**Supplementary Figure 5: 3D reconstruction of mitochondrial morphology in epithelial cells**

HCT8 cells were exposed to the vehicle control (5% LB) or bacteria-free supernatant (5%) from the wildtype *B. cereus* (75/95) for 30 minutes. Mitochondria were detected using an anti-Tom20 antibody (green), and nuclei were counterstained with DAPI (blue). White arrowheads indicate healthy tubular mitochondria, whereas orange arrowheads indicate round, fragmented mitochondria.

**Supplementary Figure 6: NheABC contributes to the metabolic paralysis of epithelial cells**

**(A)** DLD1 cells were infected with the wild-type *B. cereus* (75/95) or the mutant (75/95Δ*nheBC*) strain at an MOI, 1:50 for 4 hours at 37°C, followed by staining with TMRM (red), and Hoechst 33342 (blue). Arrowheads (white) indicate loss of mitochondrial membrane potential. Scale bars = 20 µm. Data points in the histogram to the left represent fluorescence intensity of TMRM staining (1650 x 1650 µm^2^) from independent experiments; bar graphs show mean ± s.d. Significance was determined from replicates using a one-way analysis of variance (ANOVA) with Sidak’s post-test against the uninfected cells. **p<0.01. **(B)** The line plot displays the time-dependent kinetics of leakage from PC:PE:CL liposomes induced by bacteria-free supernatants from the wild-type *B. cereus* (75/95), compared to the liposomes exposed to supernatants from the mutant (75/95Δ*nheBC*) strain. Data are representative of two to three independent replicates.

**Supplementary Figure 7:** ***Bacillus cereus* targets the cell membrane of the infected epithelial cells in 3D spheroids**

**(A)** Schematic workflow of spheroid formation and *B. cereus* infection. **(i)** HCT116 cells were added to a 96-well plate. **(ii)** 12-24 hours after addition to the 96-well plate, HCT116 cells form a loose aggregate. **(iii)** 72 hours after seeding HCT116 cells, a compact spheroid was formed that were ready for infection experiment. **(iii-A)** The compact spheroids formed in a 96-well plate were transferred to an 8-well cover-slip bottom chamber slide, followed by infection with B. cereus for 4 hours and confocal microscopy. **(iv)** Infection of HCT116 spheroids with *B. cereus* in a 96-well plate. **(B)** Membrane permeabilization of HCT116 spheroids. Spheroids cultured for 72 hours were infected with wild-type *B. cereus* (75/95) for 4 hours in an antibiotic-free medium at 37°C, followed by staining with propidium iodide (red) and Hoechst 33342 (blue). Scale bars = 100 µm. **(C)** The line graph shows the time-dependent increase in propidium iodide fluorescence in spheroids infected with the wild-type *B. cereus* (75/95) strain compared to those exposed to the mutant (75/95Δ*nheBC*) strain or the vehicle control (PBS). The line graph shows data from three independent spheroids; line plot shows mean ± s.d. **(D)** The HCT116 spheroids formed in a 96-well plate for 72 hours were transferred to an 8-well glass bottom slide and infected with the wild-type *B. cereus* (75/95) for 4 hours. The spheroids were stained with propidium iodide (red), Hoechst 33342 (blue), and FITC-Dextran (yellow). The line plot to the right indicates an increase in the number of propidium iodide positive cells and an increase in the penetration of FITC-Dextran into the lumen of the infected spheroid, compared to the uninfected spheroid. Scale bars = 200 µm.

**Supplementary Figure 8:** ***B. cereus* supernatant induces dose-dependent lysis of human erythrocytes.**

**(A)** A turbidity assay was used to determine the kinetics of erythrocyte lysis by measuring the optical density of a human erythrocyte suspension every 10 minutes following the addition of increasing concentrations of bacteria-free supernatants collected from the wild-type *B. cereus* (75/95) over a time-period of 14 hours at 37°C. LB (10% v/v) was used as vehicle control. **(B)** The t_1/2_ of the decrease in erythrocyte turbidity was reduced by approximately three-fold when erythrocytes were exposed to the 10% bacteria-free supernatant compared to the 5% concentration. Data points represent three replicates from independent experiments; bar graphs show mean ± s.d. Significance was determined from replicates using unpaired Student’s t-test. **p<0.01.

**Supplementary Figure 9:** **Genetic complementation of *nheBC* restores hemolytic activity of the mutant strain**

**(A)** Western blot analysis of bacteria-free supernatants collected from the *nheBC* complemented mutant (75/95Δ*nheBC*/p*nheBC*) strain. The mutant strain with the empty plasmid (pHT304) served as the vector control (75/95Δ*nheBC*/pVector). **(B)** A turbidity assay was used to determine the kinetics of erythrocyte lysis by measuring the optical density of a human erythrocyte suspension every 10 minutes following the addition of bacteria-free supernatants collected from the *nheBC* complemented strain (75/95Δ*nheBC*/p*nheBC*) or the vector control (75/95Δ*nheBC*/pVector).

**Supplementary Figure 10: Acidic pH decreases cell membrane permeabilization in response to bacteria-free supernatants**

**(A)** DLD1 cells pre-stained with Hoechst 33342 (2 µM) in RPMI-1640 medium adjusted to pH 7.4 or 5.5 were exposed to bacteria-free supernatant (5 %) collected from wild-type *B. cereus* (75/95). Images were acquired every 15 minutes post-treatment, and the number of propidium iodide positive cells were normalized against total number of Hoechst 33342 positive cells. Data in the line graph represent the mean ± s.d. from three replicates. **(B)** Representative fluorescence microscopy images at the 2-hour time point from the data presented in panel (A). Scale bars = 20 µm.

**Supplementary Figure 11:** **Unedited version of figure 6A**

**Supplementary Figure 12: Uncropped Western blots used in the current study**

**Table S1: Bacterial species used in the study**

| **Strains** | **Description/Relevant characteristics** | **Pore-forming toxins** |
| --- | --- | --- |
| *Escherichia coli* 536 | UPEC strain (O6:K15:H31) wild type ^1^ | HlyA (β-PFT) ^1^ |
| *Yersinia enterocolitica* | Wild type (W22703) | YaxAB (α-PFT) ^2^ |
| *Photorhabdus luminescens* | Wild type (TT01) ^3^ | XaxAB-like toxin (α-PFT) ^3^ |
| *Serratia marcescens* | Wild type (ATCC 274) | SmhABC, (α-PFT) ^4^ |
| *Vibrio cholerae* A1552 | Serogroup O1, RifR ^5^ | MakABE (α-PFT) ^6^, VCC (β-PFT) |
| *Aeromonas hydrophila* AH3 | Serotype O:34^7^ | AhlABC (α-PFT) ^8^, aerolysin (β-PFT) |
| *Bacillus cereus* | Wild type (NVH0075/95) ^9^ | NheABC (α-PFT) ^10^ |
| *Bacillus cereus ΔnheBC* mutant | *ΔnheBC* (NVH0075/95) ^11^ | None |
| *Bacillus subtilis* 168 | BGSC, Bacillus Genetic Stock Centre, Columbus^12^ | None |
| *Bacillus cereus* Δ*nheBC*::pHT304 | Vector control | None |
| *Bacillus cereus* Δ*nheBC*::pHT304*-nheBC* | NheBC complemented strain | NheABC (α-PFT) |

**References**

(1) Berger, H.; Hacker, J.; Juarez, A.; Hughes, C.; Goebel, W. Cloning of the chromosomal determinants encoding hemolysin production and mannose-resistant hemagglutination in Escherichia coli. *J Bacteriol* **1982**, *152* (3), 1241-1247. DOI: 10.1128/jb.152.3.1241-1247.1982.

(2) Wagner, N. J.; Lin, C. P.; Borst, L. B.; Miller, V. L. YaxAB, a Yersinia enterocolitica pore-forming toxin regulated by RovA. *Infect Immun* **2013**, *81* (11), 4208-4219. DOI: 10.1128/IAI.00781-13 From NLM Medline.

(3) Zhang, X.; Hu, X.; Li, Y.; Ding, X.; Yang, Q.; Sun, Y.; Yu, Z.; Xia, L.; Hu, S. XaxAB-like binary toxin from Photorhabdus luminescens exhibits both insecticidal activity and cytotoxicity. *FEMS Microbiol Lett* **2014**, *350* (1), 48-56. DOI: 10.1111/1574-6968.12321 From NLM Medline.

(4) Churchill-Angus, A. M.; Schofield, T. H. B.; Marlow, T. R.; Sedelnikova, S. E.; Wilson, J. S.; Rafferty, J. B.; Baker, P. J. Characterisation of a tripartite alpha-pore forming toxin from Serratia marcescens. *Sci Rep* **2021**, *11* (1), 6447. DOI: 10.1038/s41598-021-85726-0.

(5) Yildiz, F. H.; Schoolnik, G. K. Role of rpoS in stress survival and virulence of Vibrio cholerae. *J Bacteriol* **1998**, *180* (4), 773-784. DOI: 10.1128/JB.180.4.773-784.1998.

(6) Nadeem, A.; Nagampalli, R.; Toh, E.; Alam, A.; Myint, S. L.; Heidler, T. V.; Dongre, M.; Zlatkov, N.; Pace, H.; Bano, F.; et al. A tripartite cytolytic toxin formed by Vibrio cholerae proteins with flagellum-facilitated secretion. *Proc Natl Acad Sci U S A* **2021**, *118* (47). DOI: 10.1073/pnas.2111418118.

(7) Merino, S.; Aguilar, A.; Nogueras, M. M.; Regue, M.; Swift, S.; Tomas, J. M. Cloning, sequencing, and role in virulence of two phospholipases (A1 and C) from mesophilic Aeromonas sp. serogroup O:34. *Infect Immun* **1999**, *67* (8), 4008-4013. DOI: 10.1128/IAI.67.8.4008-4013.1999.

(8) Wilson, J. S.; Churchill-Angus, A. M.; Davies, S. P.; Sedelnikova, S. E.; Tzokov, S. B.; Rafferty, J. B.; Bullough, P. A.; Bisson, C.; Baker, P. J. Identification and structural analysis of the tripartite α-pore forming toxin of Aeromonas hydrophila. *Nature Communications* **2019**, *10* (1), 2900. DOI: 10.1038/s41467-019-10777-x.

(9) Lund, T.; Granum, P. E. Characterisation of a non-haemolytic enterotoxin complex from Bacillus cereus isolated after a foodborne outbreak. *FEMS Microbiol Lett* **1996**, *141* (2-3), 151-156. DOI: 10.1111/j.1574-6968.1996.tb08377.x.

(10) Lindback, T.; Fagerlund, A.; Rodland, M. S.; Granum, P. E. Characterization of the Bacillus cereus Nhe enterotoxin. *Microbiology (Reading)* **2004**, *150* (Pt 12), 3959-3967. DOI: 10.1099/mic.0.27359-0 From NLM Medline.

(11) Fagerlund, A.; Lindback, T.; Storset, A. K.; Granum, P. E.; Hardy, S. P. Bacillus cereus Nhe is a pore-forming toxin with structural and functional properties similar to the ClyA (HlyE, SheA) family of haemolysins, able to induce osmotic lysis in epithelia. *Microbiology (Reading)* **2008**, *154* (Pt 3), 693-704. DOI: 10.1099/mic.0.2007/014134-0.

(12) Spizizen, J. Transformation of Biochemically Deficient Strains of Bacillus Subtilis by Deoxyribonucleate. *Proc Natl Acad Sci U S A* **1958**, *44* (10), 1072-1078. DOI: 10.1073/pnas.44.10.1072.
